# Leucine regulates autophagy via acetylation of the mTORC1 component raptor

**DOI:** 10.1038/s41467-020-16886-2

**Published:** 2020-06-19

**Authors:** Sung Min Son, So Jung Park, Eleanna Stamatakou, Mariella Vicinanza, Fiona M. Menzies, David C. Rubinsztein

**Affiliations:** 1 Department of Medical Genetics, Cambridge Institute for Medical Research (CIMR), University of Cambridge, Cambridge, UK; 2 UK Dementia Research Institute, Cambridge Institute for Medical Research (CIMR), University of Cambridge, Cambridge, UK

**Keywords:** Macroautophagy, Nutrient signalling, Acetylation

## Abstract

Macroautophagy (“autophagy”) is the main lysosomal catabolic process that becomes activated under nutrient-depleted conditions, like amino acid (AA) starvation. The mechanistic target of rapamycin complex 1 (mTORC1) is a well-conserved negative regulator of autophagy. While leucine (Leu) is a critical mTORC1 regulator under AA-starved conditions, how Leu regulates autophagy is poorly understood. Here, we describe that in most cell types, including neurons, Leu negatively regulates autophagosome biogenesis via its metabolite, acetyl-coenzyme A (AcCoA). AcCoA inhibits autophagy by enhancing EP300-dependent acetylation of the mTORC1 component raptor, with consequent activation of mTORC1. Interestingly, in Leu deprivation conditions, the dominant effects on autophagy are mediated by decreased raptor acetylation causing mTORC1 inhibition, rather than by altered acetylation of other autophagy regulators. Thus, in most cell types we examined, Leu regulates autophagy via the impact of its metabolite AcCoA on mTORC1, suggesting that AcCoA and EP300 play pivotal roles in cell anabolism and catabolism.

## Abstract (web summary)

Leucine is a critical amino acid that inhibits autophagy. Here, the authors show that the leucine inhibits autophagy in most cell types, predominantly via its catabolite acetyl CoA, which drives acetylation of raptor, which activates mTORC1, a negative regulator of this catabolic process.

## Introduction

Macroautophagy (“autophagy”) is a major catabolic process that involves the highly regulated sequestration of intracytoplasmic contents in double-membrane vesicles, called autophagosomes, which later fuse with lysosomes, resulting in the degradation of the inner autophagosomal membrane and luminal content^1^. In mammalian cells, autophagosomes are formed from precursor membrane structures that include a complex of the autophagic proteins ATG5, ATG12, and ATG16L1. The phagophore membranes expand to fully enclose its cargo, forming the autophagosomes, and the completed autophagosomes traffic along microtubules to enable fusion with the lysosome^2,3^. Autophagosome–lysosome fusion results in autolysosomes that enable the enzymatic degradation of the cargo and subsequent release of recycled building blocks from macromolecules, which protects cells against starvation and related stresses.

Nutrient starvation induces autophagy in eukaryotic cells through inhibition of the evolutionarily conserved mechanistic target of rapamycin (mTOR) complex 1 (mTORC1)^4^. mTORC1, as a central regulator of cell growth^5^, plays a key regulatory role coordinating the balance between cell growth and catabolism (via autophagy) in response to nutritional status and a variety of stress signals. Amino acids (AAs), like leucine (Leu), signal to mTORC1 through RRAG GTPases, which results in the induction of mTORC1 activity by its translocation to lysosomes where it encounters RHEB, a potent mTORC1 activator^6,7^. AA starvation stimulates autophagy in cells via mTORC1 inhibition, which reduces inhibitory phosphorylation enabling activation of its substrates, including the Unc-51-like autophagy-activating kinase 1 (ULK1)^8^.

**Fig. 1:**
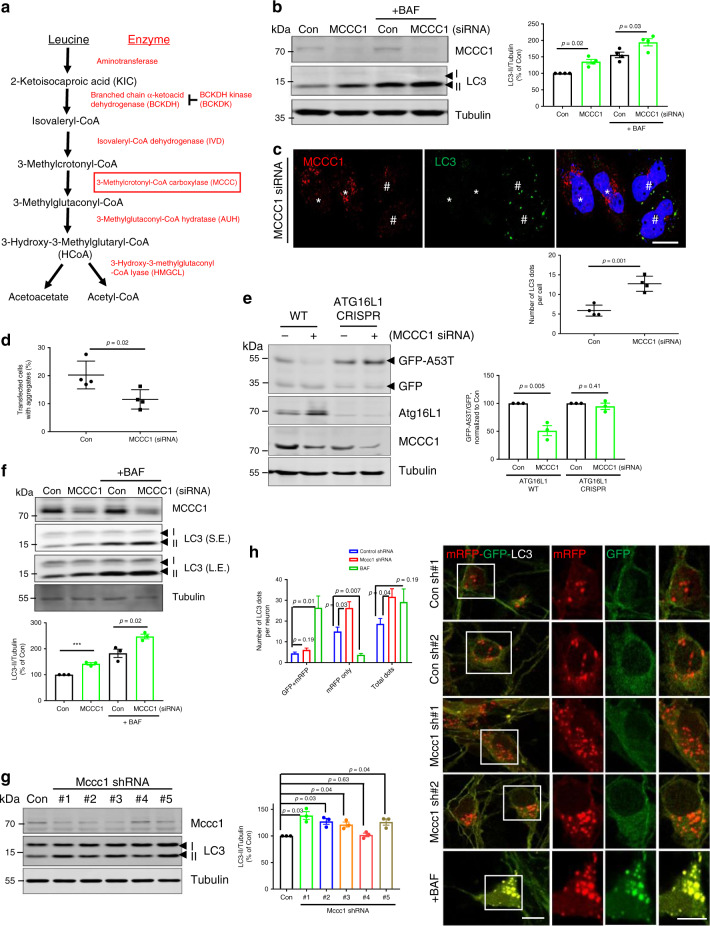
MCCC1 negatively regulates autophagy. **a** Leu metabolic pathway. Red box shows MCCC1 gene. **b** Autophagy activation by MCCC1 depletion. siRNA knockdown of MCCC1 in HeLa cells was used to determine whether MCCC1 can regulate autophagy (Con; scrambled, nontargeting siRNA, MCCC1; SMARTpool MCCC1 targeted siRNA). Blots are representative of four independent experiments (*N* = 4). Two-tailed unpaired *t*-test. **c** Immunostaining of HeLa cells treated with MCCC1 SMARTpool siRNA using MCCC1 (red) and LC3 (green) antibodies, nuclei are stained with DAPI (blue). ^#^MCCC1 knockdown cells, *non-knockdown cells. Scale bar, 10 μm. *N* = 4; 70–80 cells scored per condition per experiment. Two-tailed unpaired *t*-test. **d** Reduced HTT (Q74) aggregation in MCCC1 knockdown HeLa cells. HeLa cells were seeded on coverslips in triplicates, transfected with siRNAs targeting control or MCCC1, followed by HA-tagged HTT (Q74) expression. *N* = 4, 40–60 cells scored per condition per experiment. Two-tailed unpaired *t*-test. **e** α-synuclein degradation assay in control or MCCC1 knockdown ATG16L1 WT or CRISPR KO HeLa cells. Cells were transfected with control or MCCC1 siRNAs for 3 days. In the last 24 h, cells were transfected with empty pEGFP (as a “transfection/loading control”) + pEGFP-α-synuclein A53T. Levels of α-synuclein A53T are expressed as a ratio to GFP. *N* = 3. *p < 0.05 vs. control cells; two-tailed unpaired *t*-test. Autophagy activation by MCCC1 depletion in SH-SY5Y cells (**f**) and primary neurons (**g**). *N* = 3. ***p < 0.001 vs. control cells; two-tailed unpaired *t*-test. Long exposure (LE); Short exposure (SE). **h** Autophagic flux in mouse primary cortical neurons from mRFP-GFP-LC3 (tfLC3) transgenic mice. Representative confocal z-stack images (right panel) and total number of GFP/mRFP dots (autophagosomes) and mRFP-only dots (autolysosomes). In total, 25–35 cells analyzed per condition per experiment; two-tailed unpaired *t*-test. Scale bar, 10 μm. Data are presented as mean values ± SEM. Source data are provided as a Source data file.

Acetyl-coenzyme A (AcCoA) is a key metabolic intermediate for bioenergetics and anabolism in mammalian cells^9^, and cytosolic AcCoA functions as the central precursor for fatty acid and cholesterol biosynthesis, as well as a key metabolic regulator of autophagy^10^. Previous studies have inferred that AcCoA regulates autophagy via acetylation of proteins within the core autophagy machinery^11–14^. AcCoA is produced in mitochondria by three major pathways: namely pyruvate decarboxylation, fatty acid oxidation, and the catabolism of branched-chain amino acids (BCAAs) such as Leu^15^. Among them, BCAAs, including Leu, are transaminated to branched-chain ketoacids, which subsequently undergo oxidative decarboxylation, eventually yielding AcCoA as the end product^16^. This reaction is catalyzed by several  specific enzymes, including branched-chain ketoacid dehydrogenase (BCKDH) complex, methylcrotonoyl-CoA carboxylase 1 (MCCC1), AU RNA binding methylglutaconyl-CoA hydratase (AUH) and 3-hydroxy-3-methylglutaryl-CoA lyase (HMGCL) (Fig. 1a)^17,18^. As the most bioactive BCAA, Leu is sufficient to maintain AcCoA levels and cytoplasmic protein acetylation during nutrient deprivation^10^. Because mitochondrial AcCoA cannot be directly transferred to the cytoplasm, it is combined with oxaloacetic acid (OAA) to form citrate, and then citrate is transported to the cytoplasm via the mitochondrial citrate transporter CIC (solute carrier family 25 member 1; SLC25A1)^15,19^. In the cytoplasm, the citrate can be converted back to AcCoA and OAA by ATP-citrate lyase (ACLY)^19,20^. Under nutrient-restricted conditions, cytosolic AcCoA also can be made from acetate by acetyl-CoA synthetase short-chain family member 2 (ACSS2)^15,21^.

Previous studies, predominantly using HEK-293 cells, suggested that Leu activates mTORC1^6^ by interacting with sensor proteins such as SESN1, 2^22^ and leucyl-tRNA synthetase (LARS)^23,24^. Interestingly, in some cell types, including HeLa cells, knockdowns of these sensors did not affect mTORC1 responses to Leu in nutrient-depleted condition^4,18^. In HeLa cells and numerous other cell lines and primary neurons, but not HEK-293T cells, we found that Leu signaled to activate mTORC1 via its metabolite AcCoA^18^, and not via Leu itself. AcCoA activates mTORC1 activity by stimulating the activity of the acetyltransferase EP300 in the cytoplasm, which results in acetylation of the mTORC1 component raptor at K1097. This acetylation event is necessary for raptor binding to RRAG proteins on the lysosome and is sufficient for consequent mTORC1 activation.

Here, we have explored how Leu regulates autophagy. We hypothesized that this may occur via AcCoA in many cell types. However, since acetylation of proteins, including BECN1 and PIK3C3, have been suggested to impact autophagy^11,12,25^, it was unclear if the effects of the Leu-AcCoA pathway would be mediated predominantly by acetylation of autophagy regulators, or via altered mTORC1 activity. Interestingly, in cells where Leu signaling to mTORC1 is AcCoA dependent, the dominant effects of Leu deprivation on autophagy are mediated by mTORC1 inhibition, rather than by altered acetylation of autophagy regulators. Thus, the mTORC1 component raptor appears to be the critical acetylation target for EP300 that regulates autophagy under many physiological conditions.

## Results

### MCCC1 negatively regulates autophagy

To determine whether Leu catabolism could regulate autophagy in HeLa cells, we knocked down MCCC1, a key enzyme in the Leu metabolic pathway (Fig. 1a)^26^, where knockdown reduces whole cell and cytoplasmic AcCoA levels^18^. MCCC1 knockdown increased autophagy levels assessed by the levels of LC3-II, a classical autophagosome marker (Fig. 1b, Supplementary Fig. 1a). When Bafilomycin A1 (BAF), which blocks LC3-II/autophagosome degradation^27^, was used, depletion of MCCC1 increased the levels of LC3-II (Fig. 1b), suggesting induction of autophagosome biogenesis after MCCC1 knockdown. When MCCC1 cDNA was transfected into MCCC1 siRNA-treated cells, LC3-II levels were normalized (Supplementary Fig. 1b). By immunostaining, we confirmed increased numbers of LC3-positive structures (autophagosomes) and WIPI2 dots (phagophores)^28^ in MCCC1 knockdown cells (Fig. 1c, Supplementary Fig. 1c). Functional autophagy appeared to be activated by MCCC1 depletion, as this perturbation reduced the percentage of cells with mutant huntingtin (HTT) aggregates (which correlate inversely with autophagy activity^29^) and lowered the levels of another autophagy substrate, A53T mutant alpha-synuclein (α-synuclein) in wild-type (WT) cells but not in autophagy-null cells (ATG16L1-deficient cells generated by CRISPR/Cas9 editing^30^)^31^ (Fig. 1d, e, Supplementary Fig. 2a, d). DAPI staining revealed a mild decrease in cell viability (more cells that failed to exclude the DAPI dye) in scramble siRNA-transfected cells expressing A53T mutant α-synuclein (*p* = 0.045), which was normalized and reduced after MCCC1 knockdown, indicating that depletion of MCCC1 rescues A53T mutant α-synuclein-induced cell toxicity (Supplementary Fig. 2a, c). In human neuroblastoma SH-SY5Y cells, like in HeLa cells, MCCC1 depletion increased LC3-II levels in both basal and BAF-treatment conditions (Fig. 1f). Likewise Mccc1 knockdown in primary murine neurons resulted in increased LC3-II levels (Fig. 1g). Next, we confirmed autophagy activation in MCCC1-depleted conditions with another autophagy flux assay in HeLa cells and primary cortical neurons of mice that transgenically express the tandem fluorescent-tagged mRFP-GFP-LC3 reporter (tfLC3) (Fig. 1h, Supplementary Fig. 3a)^32^. This tool exploits the different pKa of the two LC3 tags (~4.5 for mRFP and around 6 for GFP), so the GFP signal is rapidly quenched by the acidic lysosomal environment giving red-only autolysosomes, compared with yellow autophagosomes^32^. MCCC1 knockdown in HeLa cells and primary neurons significantly increased the numbers of functional autolysosomes (the vesicles that emit only the red signals), while BAF-treatment inhibited it, since this drug blocks lysosome acidification (Fig. 1h, Supplementary Fig. 3a). These data showed that MCCC1 depletion activates autophagy in several cell types, including HeLa and neuronal cells.

### MCCC1 regulates autophagy by perturbing Leu metabolism

**Fig. 2:**
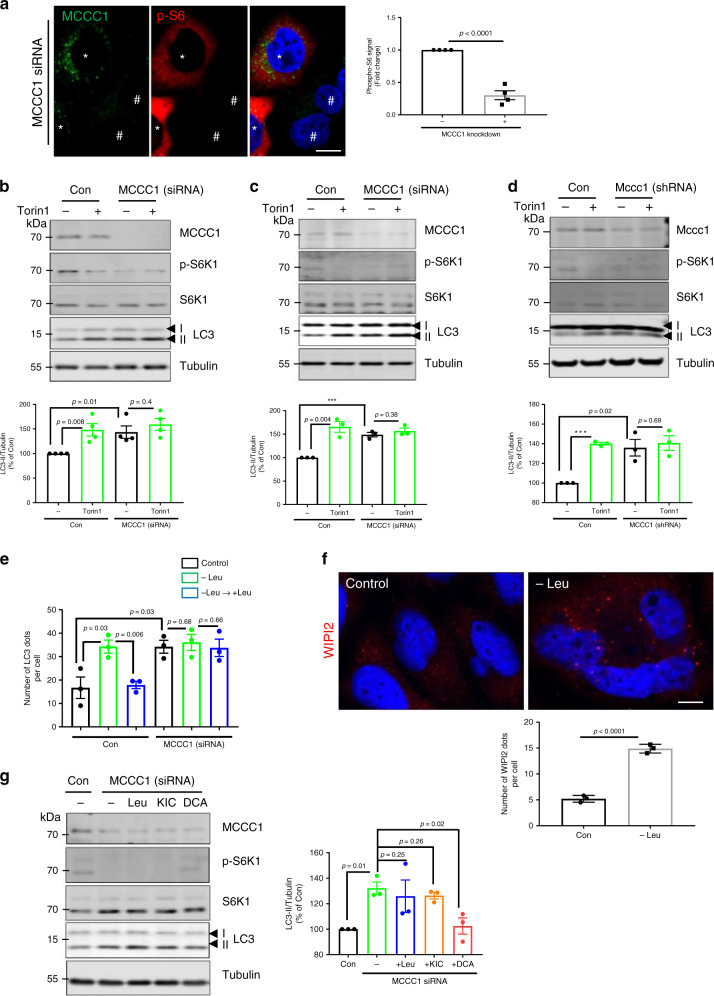
MCCC1 knockdown induces autophagy by perturbing Leu metabolism which inhibits mTORC1. **a** mTORC1 inhibition in MCCC1-depleted cells. HeLa cells were treated with MCCC1 siRNA and immunostained for MCCC1 (green) and phosphorylated S6 (red), nuclei were stained with DAPI (blue). For ease of visualization, the cell outlines are highlighted in white dashed lines. ^#^MCCC1 knockdown cells, *non-knockdown cells. Scale bar, 10 μm. *N* = 4, 30–40 cells scored per condition per experiment. Two-tailed unpaired *t*-test. The effect of mTOR inhibitor Torin1 in MCCC1 knockdown HeLa (**b**) or SH-SY5Y (**c**) and Mccc1 knockdown primary neurons (**d**). Control and MCCC1 knockdown cells were treated with 0.5 µM Torin1 (or DMSO) for 4 h. *N* = 4 for HeLa cells or *N* = 3 for SH-SY5Y and primary neurons. ***p < 0.001 vs. control cells; two-tailed unpaired *t*-test. **e** Autophagy response to changes in Leu levels. HeLa cells were treated with scrambled siRNA or MCCC1 siRNA then incubated in Leu-depleted media for 4 h or incubated in Leu-depleted media for 4 h, followed by the re-addition of 10 µM of Leu to the media for 1 h. *N* = 3, 50–60 cells scored per condition per experiment. Two-tailed unpaired *t*-test. **f** Increased number of WIPI2 dots in Leu-depleted media. *N* = 3, 40 cells scored per condition per experiment. Scale bar, 5 μm. Two-tailed unpaired *t*-test. **g** Rescue of autophagy and mTORC1 activation in MCCC1 knockdown by dichloroacetate (DCA), not Leu or KIC. MCCC1 knockdown HeLa cells were treated with 10 µM Leu, 10 mM KIC, or 10 mM DCA for 1 h. *N* = 3. Two-tailed unpaired *t*-test. Data are presented as mean values  ± SEM. Source data are provided as a Source data file.

To investigate the mechanism by which MCCC1 regulates autophagy, we confirmed our previous findings that MCCC1 inhibition impaired mTORC1 activity^18^ by demonstrating decreased staining of MCCC1 knockdown cells with an anti-phosphorylated S6 antibody (Fig. 2a), and decreased phosphorylation of the mTORC1 substrate S6K1 (which, in turn, phosphorylates S6) after MCCC1 knockdown in HeLa and SH-SY5Y cells and primary neurons, and this decreased mTORC1 activity correlated with increased autophagy (Fig. 2b–d). The levels of ULK1 phosphorylated at the mTORC1 site S757^8^ were also decreased in MCCC1 knockdown cells (Supplementary Fig. 4a)—phosphorylation of ULK1, a key autophagy protein, at this site inhibits autophagosome biogenesis. MCCC1 knockdown did not further increase LC3-II levels mediated by the mTOR inhibitors Torin1 and rapamycin^33^, compared with the clear induction caused by MCCC1 depletion in control cells (Fig. 2b–d, Supplementary Fig. 4b), suggesting that reduced MCCC1 activates LC3-II by regulating mTORC1 activity.

Because MCCC1 regulates Leu metabolism^26^ and Leu is the most bioactive AA that regulates mTORC1 signaling^6^, we next investigated whether autophagy regulation by MCCC1 is mediated via this pathway. In GFP-LC3 stably expressing HeLa cells, Leu depletion increased LC3 dot numbers and restimulation with Leu normalized this effect (Fig. 2e). However, in cells with MCCC1 knockdown, there were increased numbers of GFP-LC3 structures compared with control cells, and no significant changes were observed with Leu depletion or Leu replenishment of Leu-depleted cells (Fig. 2e). The number of WIPI2 puncta was significantly increased by Leu depletion (Fig. 2f) supporting activation of autophagosome biogenesis by Leu depletion. Consistent with our earlier results^18^, neither Leu nor its direct metabolite alpha-ketoisocaproate (KIC), which are both upstream of MCCC1 (see Fig. 1a), rescued the inhibition of mTORC1 activity or normalized the increase in LC3-II levels caused by MCCC1 knockdown, while dichloroacetate (DCA), which forms AcCoA via a distinct pathway, increased mTORC1 activity and normalized LC3-II levels (Fig. 2g). Thus, in HeLa cells, the effects of Leu on autophagy require the activity of the Leu-metabolizing enzyme MCCC1. Mutations in the MCCC1 gene (e.g., R385S) cause 3-methylcrotonyl-CoA carboxylase deficiency (MCCD), an autosomal recessive disorder of Leu catabolism^26^. Re-expression of WT MCCC1, but not mutant R385S MCCC1 (RS), in MCCC1 knockdown cells, normalized the increased LC3-II levels and number of LC3 dots and increased the S6K1 phosphorylation/mTORC1 activity (Supplementary Fig. 4c, d).

Knockdown of other enzymes regulating AcCoA formation from Leu had consistent effects with our model: knockdown of AU RNA binding AUH^34^ increased LC3-II and LC3 vesicle numbers, while knockdown of branched-chain ketoacid dehydrogenase kinase (BCKDK)^35^, which inhibits BCKDH, decreased LC3-II (Supplementary Fig. 5a–c). Our data with AUH knockdown suggested that these effects were associated with mTORC1 inhibition and that AUH knockdown did not further inhibit mTORC1 or increase in LC3-II in Torin1-treated cells, while such effects were seen in control cells (Supplementary Fig. 5a). As we observed with MCCC1, AUH knockdown, depletion of HMGCL (the enzyme immediately downstream of MCCC1 in the pathway from Leu to AcCoA; Fig. 1a) increased LC3-II levels and numbers of LC3 dots (Supplementary Fig. 5d, e), and Leu treatment failed to rescue LC3-II levels in Leu depleted, HMGCL knockdown cells (Supplementary Fig. 5d). Thus, these data indicate that Leu regulates autophagy and mTORC1 via a downstream metabolite and not via Leu itself.

Previous studies, predominantly using HEK-293 cells, suggested that Leu activates mTORC1 by interacting with sensor proteins such as SESN1, 2 and LARS^22,23^. To determine whether autophagy regulation by Leu metabolism is modulated by Leu sensors in HeLa cells, we knocked down SESN1, 2 or LARS in these cells. We found that Leu-mediated autophagy regulation is regulated independently by Leu sensors in HeLa cells (Supplementary Fig. 6a), consistent with our previous data with mTORC1^18^. These data support that Leu metabolism can regulate autophagy levels by modulating mTORC1. While the increase in LC3-II levels after Leu depletion appears to be less in SESN1, 2 double-knockdown cells compared with control, this is likely attributable to the reduction of autophagy seen after SESN2 knockdown that is dependent on the residual levels^36,37^ of its target gene, p53^38^.

### AcCoA is essential for Leu metabolism-mediated autophagy

**Fig. 3:**
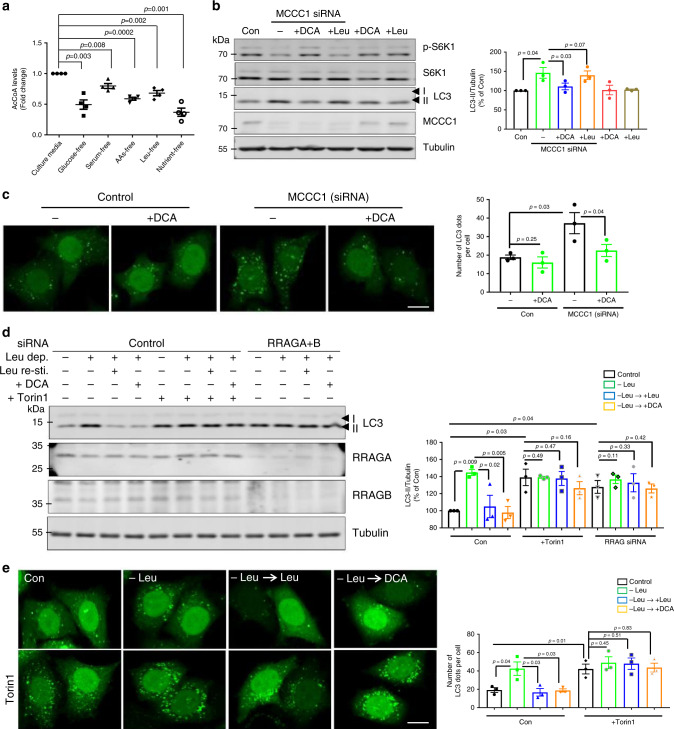
AcCoA is essential for Leu metabolism-mediated autophagy regulation. **a** AcCoA levels were assessed using the PicoProbe AcCoA assay kit. HeLa cells were incubated in nutrient-depleted conditions for 4 h followed by lysis and measurement of AcCoA levels. *N* = 4. Two-tailed unpaired *t*-test. **b** Effect of DCA and Leu on mTOR signaling and autophagy modulation caused by MCCC1 knockdown. Control cells, and cells treated with MCCC1 siRNA were treated with 10 mM DCA or 10 µM Leu for 1 h. Cells were lysed and western blots for phosphorylated S6K1 (p-S6K1) and total S6K1, as well as LC3 and MCCC1 are shown. *N* = 3. Two-tailed unpaired *t*-test. **c** Rescue of activated autophagy in MCCC1-depleted cells by DCA. 10 mM DCA for 1 h treatment restored the increased LC3 dots in MCCC1-depleted cells. *N* = 3, 60–70 cells scored per condition per experiment. Two-tailed unpaired *t*-test. Scale bar, 10 μm. AcCoA-induced autophagy regulation in mTORC1-dependent manner. Treatment with 0.5 µM Torin1 for 4 h or RRAGA and RRAGB double-knockdown (RRAGA + B) blocked the rescue effect of Leu or DCA on autophagy activation (LC3-II level (**d**) and LC3 dots (**e**)) by Leu depletion. HeLa cells were treated with scrambled siRNA or MCCC1 siRNA then incubated in Leu-depleted media for 4 h or incubated in Leu-depleted media for 4 h, followed by the re-addition of 10 µM of Leu or 10 mM DCA to the media for 1 h. *N* = 3, 40–50 cells scored per condition per experiment. Two-way ANOVA with post-hoc Tukey’s multiple comparison test. Scale bar, 10 μm. Data are presented as mean values ± SEM. Source data are provided as a Source data file.

Because AcCoA, the final Leu metabolite, is known to regulate mTORC1 and autophagy^10,18^ and we previously found decreased AcCoA levels in MCCC1 knockdown cells^18^, we determined whether autophagy regulation by Leu metabolism was mediated by AcCoA. Total AcCoA levels were reduced after depleting diverse nutrients, including Leu (Fig. 3a). Interestingly, AcCoA levels were reduced after 4 h of Leu depletion. However, after 12 h Leu depletion, AcCoA levels recovered to normal (Supplementary Fig. 7a). Consistent with an inhibitory effect of AcCoA on autophagy, LC3-II levels decreased back to normal from 4 to 12 h after Leu depletion (Supplementary Fig. 7b). It is possible that long periods of Leu depletion may activate compensatory pathways for the generation of AcCoA. When we examined the effects of different concentrations of Leu added to cells after 4 h Leu depletion, we found that 10 µM was sufficient to significantly rescue decreased mTORC1 activity, increased LC3-II levels (Supplementary Fig. 7c, e), and elevate AcCoA levels, compared with the Leu-starved cells (Supplementary Fig. 7d, e). Among BCAAs, Isoleucine (Ile), but not valine (Val), are also able to generate AcCoA via their metabolic pathways^39^. Ile, not Val, could normalize the increased LC3-II levels in MCCC1 knockdown cells (Supplementary Fig. 7f), consistent with autophagy regulation by AcCoA in MCCC1 knockdown cells and our previous data showing that high concentrations of Ile, but not Val, could rescue the decreased mTORC1 activity in AAs-starved cells^18^. DCA, which increases AcCoA levels by activating pyruvate dehydrogenase to produce AcCoA from pyruvate rather than Leu^10^, rescued the inhibition of mTORC1 activity and normalized the increase in LC3-II levels caused by MCCC1 knockdown (Figs. 2g and 3b, c). Cytosolic AcCoA also can be made by ACSS2^15^. Exogenous acetate normalized the increased LC3-II levels in Leu-depleted HeLa (Supplementary Fig. 7g, h) and SH-SY5Y cells (Supplementary Fig. 7i, j). Likewise, the increased number of LC3 dots in MCCC1 knockdown cells was normalized by acetate treatment (Supplementary Fig. 7k).

Next, we investigated whether autophagy regulation by AcCoA in Leu-depleted conditions was mediated by mTORC1. This question is critical, as the literature suggesting that acetylation of mammalian ATG proteins inhibits autophagy provides an alternative model for the effects of AcCoA and acetylation on autophagy that is independent of mTORC1^3,12,13,40,41^. While DCA (AcCoA) inhibited autophagy in Leu-depleted cells, Torin1 treatment mimicked the elevation of LC3-II seen in Leu-depleted conditions and neither addition of Leu or DCA to such Torin1-treated cells caused a reduction of LC3-II levels and numbers of LC3-positive vesicles in the presence of Torin1 (Fig. 3d, e). Since Rag GTPases recruit mTORC1 to lysosomes for its activation^42^, we assessed if the RRAG complex influenced mTORC1 regulation and autophagy by DCA/AcCoA. RRAGA and RRAGB double-knockdown (which suppress mTORC1 activity) abrogated the ability of DCA to decrease the autophagy activation in Leu-depleted conditions (Fig. 3d). Since DCA cannot inhibit LC3-II levels in cells with mTORC1 inhibition mediated by either Torin1 or RRAGA and RRAGB double-knockdown, this suggests that AcCoA cannot reduce autophagy in mTORC1-inhibited cells.

Recently, we reported that mTORC1 regulation by Leu metabolism is cell-type specific^18^. We observed autophagy inhibition by DCA/AcCoA in most cell types after Leu depletion but less obvious effects were seen in HEK-293T and Huh7 cells (Supplementary Fig. 8a), even though Leu restimulation can normalize activated autophagy in Leu-depleted HEK-239T cells (Supplementary Fig. 8b, c), suggesting that most, but not all cells tested show decreased autophagy after starvation when treated with Leu or DCA (which forms AcCoA via a Leu-independent route).

### EP300 inhibition deacetylates raptor to activate autophagy

**Fig. 4:**
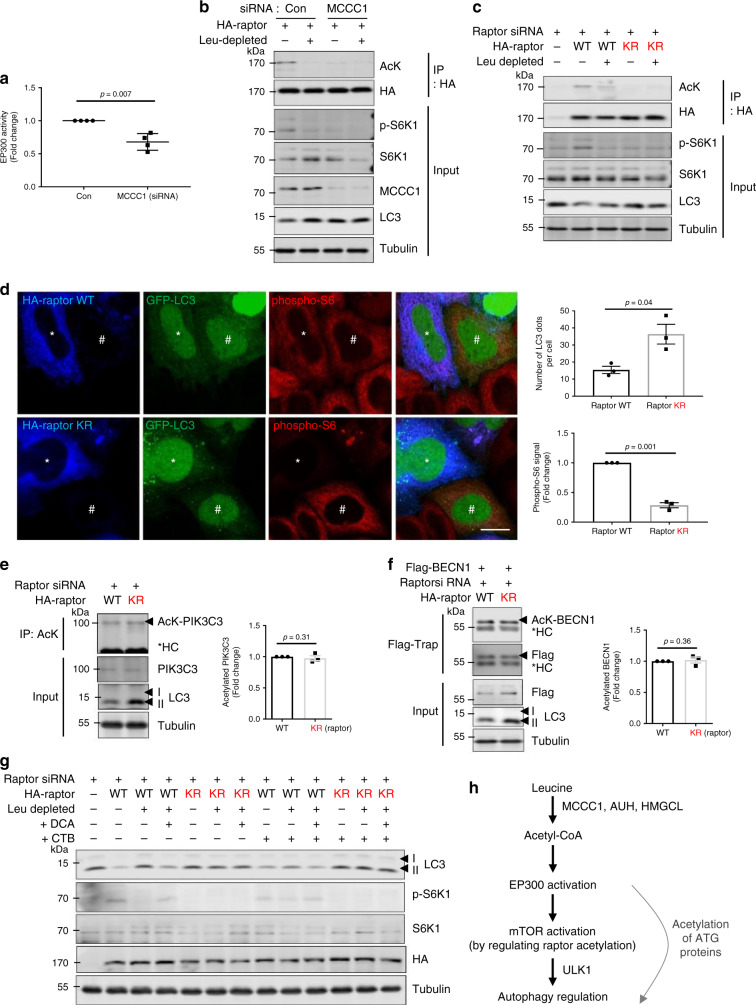
Raptor deacetylation by EP300 inactivation mediates autophagy activation after Leu deprivation. **a** HeLa cells were treated with control siRNA or MCCC1 siRNAs, and cytosolic EP300 activity was assessed. *N* = 4. Two-tailed unpaired *t*-test. **b** Reduced raptor acetylation in MCCC1 knockdown cells. HeLa cells were treated with control or MCCC1 siRNA and transfected with HA-raptor. Following incubation in Leu-depleted media for 4 h, cells were lysed and raptor was immunoprecipitated using an anti-HA antibody. *N* = 4. **c** HeLa cells were depleted of raptor with siRNA and transfected with cDNA constructs encoding either raptor WT or raptor K1097R mutant (KR) (both HA-tagged). *N* = 4. **d** mTORC1 activity (phosphorylated S6) and LC3 dot numbers in HeLa cells expressing raptor WT or raptor KR. *HA-raptor WT or KR-expressing cells, ^#^nontransfected cells. Scale bar, 10 μm. *N* = 3, 20–30 cells scored per condition per experiment. Two-tailed unpaired *t*-test. Acetylated PIK3C3 (**e**) and BECN1 levels (**f**) in HeLa cells depleted of raptor then reconstituted with raptor WT or raptor KR. Two-tailed unpaired *t*-test. HC, heavy chain. *N* = 4. **g** Effects of EP300 activator on mTORC1 signaling and autophagy in response to Leu deprivation with or without DCA in HeLa cells depleted of raptor then reconstituted with raptor WT or raptor KR. HeLa cells were incubated in Leu-depleted media for 4 h or incubated in Leu-depleted media with Torin1 for 4 h, followed by re-addition of 10 mM DCA to the media for 1 h. 50 µM CTB for 12 h was used to activate EP300. Blots are representative of three biologically independent experiments (*N* = 3). **h** Leu abundance regulates autophagy in many cell types via its metabolite AcCoA. AcCoA activates EP300, which acetylates raptor, leading to mTORC1 activation, which inhibits autophagy. In Leu deprivation conditions, autophagy activation is mainly mediated by decreased raptor acetylation causing mTORC1 inhibition, rather than by altered acetylation of other autophagy regulators. Data are presented as mean values ± SEM. Source data are in Source data file.

To investigate how lowered AcCoA levels resulting from Leu depletion or MCCC1 knockdown upregulates autophagy, we measured cytosolic AcCoA and citrate levels in Leu-depleted conditions. Total and cytosolic AcCoA and citrate levels were reduced after depleting Leu (Supplementary Fig. 9a, b), and chemical inhibition of the mitochondrial citrate carrier (which transports citrate from the mitochondria to the cytosol to be converted to AcCoA by ACLY) increased LC3-II levels in HeLa and SH-SY5Y cells (Supplementary Fig. 9c, d), suggesting that depletion of Leu activates LC3-II by regulating cytosolic AcCoA levels. Recently, we reported that AcCoA, which can be formed from Leu, stimulated the acetyltransferase EP300, and this, in turn, led to raptor acetylation that caused mTORC1 tethering to lysosomes and activation^18^. Consistent with our earlier work, EP300 activity in cytosolic fractions was reduced in MCCC1 knockdown cells (Fig. 4a) and by Leu depletion (Supplementary Fig. 10a). The specific EP300 inhibitor c646 (validated in Supplementary Fig. 10a) inhibited mTOR translocation to lysosomes (which is known to lead to impaired mTORC1 activity^6,43^), and increased LC3-II levels, whereas, its activator, CTB (validated in Supplementary Fig. 10a) decreased LC3-II (Supplementary Fig. 10b, c). As expected, c646 decreased the acetylation of PIK3C3 (VPS34), while CTB increased the acetylation of this substrate, thus validating the effects of these tools (Supplementary Fig 10c). In addition, EP300 knockdown or chemical inhibition prevented autophagy inhibition by Leu restimulation or DCA treatment in Leu-depleted cells (Supplementary Fig. 10d), suggesting that Leu and DCA are regulating autophagy in this context via EP300 activity. MCCC1 knockdown cells or cells incubated in Leu-depleted media had decreased raptor acetylation (Fig. 4b, c), as predicted^18^. The raptor acetylation appeared to be essential for regulating autophagy under Leu-depleted conditions, since cells expressing raptor KR (acetylation dead mutant (K1097R; KR)^18^) had increased LC3-II levels that were not further increased by Leu removal (Fig. 4c, d). As predicted, c646 inhibited the acetylation of raptor and the inhibition of autophagy after addition of Leu to Leu-depleted cells (Supplementary Fig. 10e). Also, DCA or acetate rescued the decreased raptor acetylation caused by Leu depletion (Supplementary Fig. 10f). Together, these data argue that the effects of Leu and AcCoA on autophagy in these paradigms are dependent on EP300 activity and raptor acetylation, the latter, which directly regulates mTORC1 tethering to lysosomes and mTORC1 activity^18^.

EP300 acetylates several substrates including autophagy-related (ATG) proteins and these events have been proposed to regulate autophagy^12^. We found that raptor KR-expressing cells did not have altered levels of acetylated autophagy-related proteins (PIK3C3 and ATG6/BECN1)^11,25^, despite having increased LC3-II levels (Fig. 4e, f). Thus, decreased mTORC1 activity does not reduce the acetylation of these autophagy-related proteins. To test if EP300-driven acetylation of proteins (besides raptor) could inhibit autophagy in Leu-depleted cells, we activated EP300 with CTB in raptor WT- and raptor KR-expressing (nonacetylatable raptor) cells. While CTB and DCA dramatically reduced LC3-II levels in Leu-depleted cells with WT raptor (lane 3 c.f. lane 9 in Fig. 4g), neither this EP300 activator nor DCA had discernable effects on LC3-II levels in cells expressing raptor KR, which leads to mTORC1 inhibition (lane 12 c.f. lane 13 in Fig. 4g). These data suggest that EP300 activation in mTORC1-inhibited cells has no obvious inhibitory effects on autophagy. Likewise, CTB did not cause any change in the increased LC3-II and did not alter the decreased mTORC1 activity in Torin1-treated cells (Supplementary Fig. 10g). Thus, EP300-dependent acetylation of proteins other than raptor does not override the autophagy induction mediated by mTORC1 inhibition.

A recent study showed that phosphorylation of EP300 by mTORC1-inhibited autophagy^44^. Thus, we assessed mTORC1 activity and autophagy in HeLa cells expressing EP300 4SA (phospho-dead) or EP300 4SD (phospho-mimetic) mutants at the EP300 residues reported to be phosphorylated by mTORC1^44^. As we observed in WT HeLa cells, Leu and DCA rescued the inhibition of mTORC1 and autophagy activation resulting from Leu depletion in EP300 4SA or 4SD mutant-expressing HeLa cells (Supplementary Fig. 11a). In addition, Leu and DCA could reverse raptor deacetylation by Leu depletion in EP300 4SA or 4SD mutant-expressing HeLa cells (Supplementary Fig. 11b). Thus, in HeLa cells, these proposed phosphorylation sites do not impact mTORC1 regulation via the Leu-AcCoA pathway.

Thus, our data argue that the overwhelming effect of Leu on starvation-induced autophagy is mediated by raptor acetylation, which activates mTORC1. Previously, we found reduced mTORC1 activity, AcCoA levels and significantly decreased levels of acetylated raptor in fasted mouse tissues^18^. With these same samples, we found increased LC3-II levels in the brains and muscles of fasted compared with fed mice (Supplementary Fig. 11c), and observed significant correlations between AcCoA levels and acetylated raptor levels, and negative correlations between AcCoA and LC3-II, and acetylated raptor and LC3-II (Supplementary Fig. 12a–f), compatible with our cell culture data.

## Discussion

Our data suggest that Leu abundance regulates autophagy in many cell types via the Leu metabolite AcCoA, and not via Leu itself. AcCoA activates EP300, which acetylates raptor, leading to mTORC1 activation, which inhibits autophagy. These data suggest that cytoplasmic AcCoA abundance is a critical determinant of mTORC1 activity and autophagy, and will thus impact both anabolic (e.g., translation) and catabolic processes in most, but not all, cell types (Fig. 4h), including primary cells and stem cells. Our data in this paper and in our previous study^18^ show that AcCoA is both necessary and sufficient for mTORC1 activation and autophagy inhibition by Leu replenishment after AA (or Leu) depletion. Thus, the effects we have observed cannot be achieved by other metabolites alone, like α-ketoglutarate, under these conditions, as the mTORC1 activation and consequent autophagy inhibition were dependent on EP300-mediated acetylation of raptor at K1097. In other words, AcCoA is required, AcCoA induction of mTORC1 is EP300 activity dependent and can be mimicked by EP300 activation, and these mTORC1 signaling and autophagy effects are abrogated when raptor acetylation is inhibited either by EP300 inhibition or mutation of K1097 in raptor.

To determine how Leu metabolism to AcCoA occurring in the mitochondria matrix can affect EP300-mTORC1 (via raptor) and autophagy in the cytosol, we measured cytosolic citrate and AcCoA levels. Leu-derived AcCoA is converted to citrate within mitochondria to be released to the cytosol, where it is converted back to AcCoA^19,20^. Depletion of Leu decreased cytosolic citrate and AcCoA levels (Supplementary Fig. 9a, b). In addition, inhibition of the mitochondrial citrate carrier that transports citrate from mitochondria to the cytoplasm increased LC3-II levels and abrogated the normalization of LC3-II by Leu after Leu depletion (Supplementary Fig. 9c, d). These data are compatible with the understanding of how mitochondrial Leu metabolism impacts cytoplasmic AcCoA levels and EP3000 activity.

Interestingly, MCCC1, a key enzyme in the Leu-AcCoA pathway, was reported as a possible Parkinson’s disease (PD) risk locus in GWAS studies^45^. Indeed, depletion of MCCC1 enhanced the autophagic clearance of A53T mutant α-synuclein, a mutant protein that can cause PD, and ameliorated its toxicity (Supplementary Fig. 2a, c). Mutations in MCCC1 cause an autosomal recessive disease due to the loss of enzyme activity that manifests with hypoglycemia, ketonaemia, and severe metabolic acidosis^46^. It is most likely that the severe inhibition of the Leu metabolic pathway causes disease because of primary metabolic derangements, although we cannot exclude effects of mTORC1 and autophagy perturbations. However, the situation in these cases with autosomal recessive disease with minimal residual activity is likely to be very different from the effects of a GWAS-associated variant, where one would expect only very mild effects on activity and no overt selective disadvantage.

Cytosolic AcCoA can be produced in mitochondria by three major pathways, including pyruvate decarboxylation or Leu catabolism, and under nutrient-restricted conditions, cytosolic AcCoA also can be made from exogenous acetate by ACSS2. Pyruvate decarboxylation is negatively regulated by pyruvate dehydrogenase kinases (PDKs)^47^, so pharmacological inhibition of PDKs with DCA induces upregulation of whole cell and cytosolic AcCoA^10,18^. DCA reversed the increased autophagy levels (Figs. 2–4) and rescued the reduced raptor acetylation caused by Leu depletion (Supplementary Fig. 10f). Like DCA, when sodium acetate was applied to Leu-depleted media, it also reversed the increased autophagy levels (Supplementary Fig. 7g–k) and the decreased raptor acetylation resulting from Leu depletion (Supplementary Fig. 10f), suggesting that the abundance of AcCoA is a central regulator of autophagy. Interestingly, our data suggest that the AcCoA-autophagy mechanism appears to be relevant in a variety of cell types, including neurons. However, it is not obvious in HEK-293T cells or Huh7 cells (Supplementary Fig. 8). This raises the possibility of cell type-specific mechanisms regulating autophagy under nutrient-restricted conditions. There is the extensive literature showing that glucose can stimulate AcCoA levels^15,48,49^. However, glucose regulates autophagy in complex ways—glucose depletion stimulates AMPK to induce autophagy^50,51^, while paradoxically, excess glucose transport into the cells can also stimulate autophagy in an mTORC1-dependent manner^52^.

Previous papers suggested that multiple different lysine acetyltransferases (KATs) or lysine deacetylases (KDACs; referred to as HDACs) play pivotal roles in mammalian autophagy regulation at multiple steps of the pathway^53^. Here, we found that inhibition of EP300 induced autophagy, and that EP300 activation inhibited autophagy (Supplementary Fig. 10). These effects can be attributed to EP300-mediated raptor acetylation, which enables mTORC1 activation by tethering this kinase complex to lysosomes. Importantly, since addition of AcCoA (in the form of DCA) or stimulation of EP300 activity had no effects on the elevated LC3-II levels in Leu-depleted cells, acetylation of proteins other than raptor appears to have little detectable effect on autophagy in this context. Thus, this study illustrates the functional effect of the Leu-AcCoA-EP300 regulation of autophagy via mTORC1.

## Methods

### Cell culture

HeLa, SH-SY5Y, HEK-293T, Huh7, and H4 cells (from ATCC) were cultured in Dulbecco’s modified Eagle’s medium (DMEM) (4.5 g l^−1^ of glucose; #D6546) supplemented with 10% FBS (#F7524), 2 mM l-glutamine (#G7513), and 100 U ml^−1^ penicillin and 100 mg ml^−1^ streptomycin (#P0781). HeLa cells stably expressing either mRFP-GFP-LC3 (tfLC3) or GFP-LC3 were grown in the same media supplemented with 500 µg ml^−1^ of G418 (1181031 Thermo Fisher Scientific). MCF10A cells were purchased from Horizon (Catalog No. HD PAR-058) and cultured in DMEM-F12 supplemented with 5% horse serum (#H1270), 20 ng ml^−1^ hEGF (#E9644), 0.5 μg ml^−1^ hydrocortisone (#H0135), 100 ng ml^−1^ cholera toxin (#C8052), 10 μg ml^−1^ insulin (#I9278) and 100 U ml^−1^ penicillin and 100 mg ml^−1^ streptomycin (all the components were obtained from Sigma-Aldrich), as described previously^18^. A human adipose-derived Mesenchymal Stem Cells cell line was purchased from ATCC (#PCS-500-011), and maintained in Mesenchymal Stem Cell Basal Medium (ATCC, #PCS-500-030), supplemented using the Mesenchymal Stem Cell Growth Kit (ATCC, #PCS-500-040), according to the manufacturer’s recommendation. HeLa CRISPR/Cas9 ATG16L1 knockout (KO) cell lines were generated using a double-nicking strategy with paired guide RNAs to avoid off-target activity^30^. All the cell lines were maintained at 37 °C and 5% CO_2_ and were regularly tested for mycoplasma contamination using EZ-PCR Mycoplasma Detection Kit (#20-700-20 from Biological Industries). HeLa, SH-SY5Y, HEK-293T and MCF10A cells are female, and H4 cells are male. For Leu depletion to cell lines, cells were washed three times in Hank’s balanced salt solution (HBSS) (Invitrogen) and incubated for 4 h at 37 °C in Leu-free medium from Sigma-Aldrich or Crystalgen Inc. containing 10% dialyzed FBS (#26400-036 from Invitrogen) unless otherwise indicated.

### Primary mouse cortical neurons and glial cells

WT primary cortical neurons were isolated from C57BL/6 mice (Jackson Laboratories) embryos at E16.5 as described previously^54^. For primary mRFP-GFP-LC3 (tfLC3) cortical neurons, transgenic mice were crossed with C57BL/6 mice. At E16.5 gestation, females were sacrificed and embryos were harvested. Cortices from all embryos (regardless of genetic status) were combined to create mixed cultures^55^. Briefly, brains were harvested and placed in HBSS where the meninges were removed and the cerebral cortices were dissected. After incubation in HBSS with 0.25% trypsin (Gibco) for 20 min at 37 °C, dissociated neurons were resuspended in HBSS and seeded on poly-D-lysine-coated 6- or 12-multiwell plates. Cells were cultured in maintenance media (Neurobasal-A medium (#12349015, Thermo Fisher Scientific) supplemented with 2 mM GlutaMAX, 200 mM B27 supplement and 1% Penicillin–Streptomycin) at 37 °C in a humidified incubator with 5% CO_2_. One half of the culture medium was changed every 2 days until treatment/infection. After 5 days of in vitro culturing, differentiated neurons were infected with lentiviral particles for knockdown experiments. Primary glial cells were prepared as reported previously^56^.

### Animal studies

The autophagy reporter mRFP-GFP-LC3 reporter mouse line was housed in individually ventilated cages with free access to standard animal food chow and water, in a climate-controlled room with a 12 h light/dark cycle. This mouse line was generated in our lab as previously described^55^. As in our earlier study^18^, we used 6–7-weeks-old C57BL/6 male or female mice for food deprivation in vivo. The ratio of sexes of used mice was 1:1 for the 48 h fasting experiment. The number of the mice used for the experiments are indicated for each experiment in the figure legends (in general, *n* = 6). No inclusion or exclusion criteria were used. No significant differences of LC3-II levels between sexes were observed. All studies and procedures were performed under the jurisdiction of appropriate Home Office Project and Personal animal licenses and with local Ethics Committee approval.

### Antibodies and reagents

The following antibodies have been used in this work: mouse anti-Flag M2 (#F3165), rabbit anti-Actin (#A2066), and mouse anti-α-Tubulin (#T9026) from Sigma-Aldrich; mouse anti-GAPDH clone 6C5 (#ab8245), rabbit anti-MCCC1 (#ab178675), rabbit anti-SESN1 (#ab134091), rabbit anti-LC3B (#ab192890), rabbit anti-HMGCL (#ab97293), mouse anti-WIPI2 (#ab105459), rabbit anti-HMGCL (#ab97293), and rabbit anti-AUH (#ab157453) from Abcam; mouse anti-BCKDK (#NBP1-47664), goat anti-HA (#NB600-362), and rabbit anti-MCCC1 (#NBP1-81254) from Novus Biologicals; rabbit anti-EP300 (#sc-585), goat polyclonal antiraptor (#sc-27744), mouse anti-HMGCL (#sc-100548), and mouse anti-MCCC1 (#sc-365754) from SantaCruz Biotechnology; mouse anti-LC3B (#0231-100; Nanotools); rabbit anti-ATG16L1 (#PM040; MBL); mouse anti-GFP (#632375 and #632592; Clontech); mouse anti-HA.11 clone 16B12 (#MMS-101P, Covance); mouse anti-EP300 (#05-257) from Millipore; mouse anti-LAMP1 clone H4A3 (obtained from Developmental Studies Hybridoma Bank, University of Iowa); rabbit anti-LAMP1 (#9091), rabbit anti-mTOR (#2972), rabbit anti-phospho-mTOR (Ser2481; #2972), rabbit antiraptor (#2280), rabbit anti-phospho-p-S6K1 (Thr389; #9234), anti-total S6K1 (#9202), rabbit anti-phospho-S6 Ribosomal Protein (p-S6) (Ser235/236; #4856), rabbit anti-S6 Ribosomal Protein (S6) (#2217), rabbit anti-phospho-4E-BP1 (Thr37/46; #9459), rabbit anti-4E-BP1 (#9452), rabbit anti-phospho-ULK1 (Ser757; #6888), rabbit anti-ULK1 (#4773), rabbit anti-SESN2 (#8487), rabbit anti-LARS (#13868), rabbit anti-PIK3C3 (#4263), rabbit anti-acetylated-Lysine (Ac-K) (#9814, #9441), rabbit anti-RRAGA (#4357), rabbit anti-RRAGB (#8150), rabbit anti-WIPI2 (#8567), and rabbit anti-ATG16L1 (#8089) from Cell Signaling Technology; antimouse (#NA931V) and antirabbit (#NA934V) horseradish peroxidase (HRP)-conjugated secondary antibodies (GE Healthcare); antigoat HRP-conjugated secondary antibody (#611620, Invitrogen/Life Technologies). All primary antibodies were used at a dilution between 1:500 and 1:1000 (overnight incubation at 4 °C), and the secondary antibodies used at a dilution of 1:4000 (1 h of incubation at room temperature).

Drug treatments used include: DMSO, 1–100 µM Leu, 0.1–10 mM Ile, 0.1–10 mM Val, 0.1–10 mM KIC, 1–10 mM sodium DCA, 10 µM c646, 50 µM CTB, 10 mM NAM, 2 µM TSA, 10 mM sodium butyrate, 0.1–2 mM sodium acetate, 2–5 mM BTC from Sigma-Aldrich; 0.2–1 µM Torin1 from Tocris Bioscience; 0.5–2 µM rapamycin from LC Laboratories; 400 nM BAF from Enzo Life Sciences; 500 µg ml^−1^ Geneticin Selective Antibiotic (G418).

### Transfection

TransIT-2020 reagent (#MIR5400; Mirus) was used for DNA transfection, while Lipofectamine 2000 (#11668) or Lipofectamine RNAiMAX (#13778) (Invitrogen) was used for siRNA transfections, according to the manufacturer’s instructions. For knockdown experiments, cells were transfected with 20–50 nM siRNA followed by another 20–50 nM siRNA transfection after 48 h. Cells were split once between both transfections and harvested after 3 days of transfection. The following DNA or siRNA/shRNA constructs were also used in this work: empty pEGFP (Clontech), pEGFP-α-synuclein A53T and pcDNA3.1-myc-6XHis (Invitrogen); pCMV6-XL5-MCCC1 (#SC113201) from Origene; pcDNA3.1-EP300-6XHis (#23252), pRK5-HA-YFP-raptor (#73385), pRK5-HA-raptor (#8513), pcDNA4-BECN1-Flag (#24388) from Addgene. Huntington’s disease gene exon 1 fragment with 74 poly-Q repeats in pEGFP-C1 (Clontech; EGFP-HTT(Q74)) was previously characterized^57^. Predesigned siRNAs (SMARTpool and/or set of deconvoluted oligos ON-TARGETplus4Non-targeting Control #D-001810-10; RRAGA #L-016070-00-0005, RRAGB #L-012189-01-0005, AUH #L-008457-00-0005, EP300 #L-003486-00-0005, RAPTOR #L-004107-00-0005, SESN1 #L-020244-00-0005, SESN2 #L-019134-02-0005, LARS #L-010171-00-0005, HMGCL #L-019290-00-0005, BCKDK #L-004932-00-0005) were obtained from Dharmacon-Thermo Scientific; For knockdown of MCCC1, we used siRNA from Ambion (#AM51331, s32399, s32400, s32401) as well as from Dharmacon-Thermo Scientific (#L-009429-00-0005). Predesigned pLKO.1 shRNAs vectors from The RNAi Consortium (TRC) (empty vector control #RHS4080, Dharmacon; mouse Mccc1 #RMM4534-EG72039). For autophagic flux in tfLC3 neurons, we used two single shRNAs (TRCN0000112505 (#1) and TRCN0000112506 (#2)).

### Western blot analysis

Cells were washed with ice-cold PBS and directly lysed with 2× Laemmli buffer and boiled at 100 °C for 10 min or lysed with RIPA buffer (50 mM Tris-HCl pH 7.4, 150 mM NaCl, 1% NP-40, 0.5% sodium deoxycholate monohydrate, 0.1% SDS, supplemented with protease and phosphatase inhibitors cocktails (Roche)). When lysed in RIPA buffer, cells were incubated on ice for 10 min, centrifuged at 16,100 g for 10 min and protein concentration of supernatants was determined using a Bradford assay kit (Bio-Rad). Lysates were then denatured with 2× Laemmli buffer and boiled at 100 °C for 10 min, separated by SDS-PAGE, transferred onto PVDF membranes, subjected to western blot analysis, finally visualized using an ECL enhanced chemiluminescence detection kit (GE Healthcare), or with direct infrared fluorescence detection on an Odyssey Infrared Imaging System. Densitometric analysis on the immunoblots was performed using ImageJ program or IMAGE STUDIO Lite software.

### Imaging and quantification of mRFP-GFP-LC3 primary neuron

The mRFP-GFP-LC3 primary neurons were imaged at DIV9-11 using confocal microscopy (63× NA 1.4 Plan Apochromat oil-immersion lens; Carl Zeiss LSM710 and LSM780). At least 10 fields were imaged. Images were analyzed by using Zen software. The number of yellow vesicles dots (GFP-positive and RFP-positive dots; autophagosomes) and red-only vesicles (RFP-only dots; autolysosomes) were counted. A two-tailed *t*-test was used for statistical analysis.

### α-synuclein A53T assay

HeLa WT and CRISPR/Cas9 ATG16L1 KO cells were transfected with 1 µg of pEGFP-α-synuclein A53T and 0.5 µg of empty pEGFP per well of a six-well plate. Forty-eight hours after transfection, cells were lysed and used to analyze GFP-α-synuclein A53T and GFP levels by western blotting. Ratio between both signals was calculated.

### Quantification of poly-Q aggregation

HA- or EGFP-tagged HTT (Q74) aggregation were monitored with a fluorescence microscope. The counting method has been previously described^55^. When using HTT (Q74), cells were counted per coverslip and the proportion of cells with at least one aggregate was scored as a percentage of the total number of transfected cells. The experiments were performed without knowing the identity of the slides at least three times in triplicates.

### Flow cytometry

Scramble or MCCC1 siRNA-transfected HeLa cells were transfected with GFP or GFP-α-synuclein A53T. Forty-eight hours later cells were lifted and stained with 2 µg ml^−1^ DAPI (Sigma; D9542) on ice for ~10 min. Cells were then analyzed for GFP and DAPI fluorescence in an Attune NxT Flow Cytometer (ThermoFisher Scientific) using the VL1 (405 440/50) and BL1 (488 530/30) detectors. Cells were first gated on forward (FSC-A) and side scatter (SSC-A) and then for singlets (FSC-A/FSC-H). In total, 60,000 single cells were recorded. GFP + and DAPI + gates were set using untransfected and GFP-transfected cells not stained with DAPI. Analysis was performed using the FlowJo v10 software.

### shRNA lentivirus production and infection

shRNA lentiviral particles were produced and transduced following TRC protocols. Briefly, HEK-293T packaging cells growing in 100 mm dishes were transfected at 60–70% of confluence with a mix of 2.5 µg psPAX2 vector (packaging vector), 270 ng pMD2.G vector (envelope vector) and 2.7 µg hairpin-pLKO.1 vector. TransIT-LT1 (Mirus) was used as transfection reagent, according to the manufacturer’s instructions. After transfection, cells were cultured in high-serum medium (20% FBS). Cell culture medium was harvested three times for intervals of 24 h. Viral preps were then concentrated by centrifugation at 160,100 g for 90 min. For primary neurons, 20 µl of viral preps were added to the cells in the presence of 6 µg ml^−1^ polybrene (Sigma-Aldrich) and were incubated for 24 h. The medium was replaced by full medium and cells were further incubated for an additional 4 days before testing the knockdown effects.

### Mutagenesis

Mutagenesis of the human raptor, MCCC1 and EP300 was generated with QuikChange Site-Directed Mutagenesis Kit (Agilent Stratagene) according to manufacturer’s instructions. The primers for mutagenesis were designed using web-based QuikChange Primer Design program (Agilent Technologies).

The following primer sequences were used:

1. Primers used for generating raptor K1097R:
  F: 5′-CCAAATCAGCAAAATTCCTCCAGACCCTGATGGCA-3′
  R: 5′-TGCCATCAGGGTCTGGAGGAATTTTGCTGATTTGG-3′
2. Primers used for generating MCCC1 R385S:
  F: 5′-AGGATCTTCTGCATATATGCTAGCTTCGAAGGCATGG-3′
  R: 5′-CCATGCCTTCGAAGCTAGCATATATGCAGAAGATCCT-3′

The following primer sequences were used for EP300 4SA mutagenesis:

1. Primers used for generating EP300 S2271A #1:
  F: 5′-GGCTGAACAGGGGCCCCCATCTGTTGC-3′
  R: 5′-GCAACAGATGGGGGCCCCTGTTCAGCC-3′
2. Primers used for generating EP300 S2279A #2:
  F: 5′-GCCCAACCCCATGGCCCCCCAGCAGCAT-3′
  R: 5′-ATGCTGCTGGGGGGCCATGGGGTTGGGC-3′
3. Primers used for generating EP300 S2291A #3:
  F: 5′-CTTGTAGGTGTGGGGCCTGGGCCTGATTTGG-3′
  R: 5′-CCAAATCAGGCCCAGGCCCCACACCTACAAG-3′
4. Primers used for generating EP300 S2315A #4:
  F: 5′-GTGGCCGTGGAGCAGGGACAGGCTG-3′
  R: 5′-CAGCCTGTCCCTGCTCCACGGCCAC-3′

The following primer sequences were used for EP300 4SD mutagenesis:

1. Primers used for generating EP300 S2271D #1:
  F: 5′-GTTGGGCTGAACAGGGTCCCCCATCTGTTGCTGA-3′
  R: 5′-TCAGCAACAGATGGGGGACCCTGTTCAGCCCAAC-3′
2. Primers used for generating EP300 S2279D #2:
  F: 5′-ATATGCTGCTGGGGGTCCATGGGGTTGGGCTG-3′
  R: 5′-CAGCCCAACCCCATGGACCCCCAGCAGCATAT-3′
3. Primers used for generating EP300 S2291D #3:
  F: 5′-GCCTTGTAGGTGTGGGTCCTGGGCCTGATTTGGG-3′
  R: 5′-CCCAAATCAGGCCCAGGACCCACACCTACAAGGC-3′
4. Primers used for generating EP300 S2315D #4:
  F: 5′-GGCCGTGGATCAGGGACAGGCTGGGGAG-3′
  R: 5′-CTCCCCAGCCTGTCCCTGATCCACGGCC-3′

PCR products were incubated with Dpn1 restriction enzyme for 1 h and then mixed with XL-10 Gold-competent cells for transformations. DNA was extracted from colonies and sequenced by Genewiz (UK). In order to establish EP300 4SA or EP300 4SD, EP300 S2271A or S2271D mutants were used as templates to generate EP300 S2271A-S2279A or EP300 S2271D-S2279D using mutagenesis primers for S2279A or S2279D, respectively. After establishment of EP300 2SA or 2SD double mutants, EP300 S2271A-S2279A or S2271D-S2279D were sequentially mutagenised using mutagenesis primers for EP300 S2291A or S2291D. In turn, EP300 3SA or 3SD triple mutants were used for EP300 4SA (S2271A-S2279A-S2291A-S2315A) or 4SD (S2271D-S2279D-S2291D-S2315D) mutagenesis with individual mutagenesis primers EP300 S2315A or S2315D.

### Immunofluorescence

For immunofluorescence, HeLa, HEK-293T or primary neurons were fixed for 5 min with ice-cold methanol or for 10 min with 4% paraformaldehyde. Concentration of the primary and secondary antibodies is described below. The mounting solution was from Molecular Probes.

Dilution of primary antibodies. 1:300 rabbit anti-mTOR; 1:600 mouse anti-LAMP1; 1:300 rabbit anti-phospho-S6; 1:500 mouse anti-GFP; 1:200 mouse anti-MCCC1; 1:100 mouse anti-HMGCL; 1:400 rabbit anti-LC3; 1:200 rabbit anti-WIPI2; 1:1000 mouse anti-HA.11 clone 16B12.

The secondary antibodies Alexa 488, 555, 568, 594, or 647 goat antimouse, goat antirabbit or rabbit antigoat were obtained from Molecular Probes and used at 1:400. Imaging was conducted with LSM710, LSM780, or LSM880 Zeiss confocal with 63× oil-immersion lens. Colocalization was measured using Volocity software for Mander’s Overlap Coefficient (MOC) or Pearson’s correlation coefficient. These procedures were performed in a blinded fashion.

### Acetyl-coenzyme A (AcCoA) measurement

AcCoA content was determined on total or cytosolic fractions using the PicoProbe AcCoA assay kit (ab87546, Abcam) according to manufacturer’s instructions. Briefly, after deproteinization using the perchloric acid, the CoASH Quencher and Quencher remover were added into the sample to correct the background generated by free CoASH and succ-CoA. The sample was then diluted with the reaction mix, and fluorescence was measured using a plate reader and the following settings: *λ*_ex_ = 535 nm; *λ*_em_ = 587 nm. Fluorescence was measured using a Versamax Tunable microplate reader (Molecular Devices) or Spark multimode microplate reader (TECAN Trading AG, Switzerland). The acetyl-CoA standard curve was made in the range of 0–100 pM and the correlation coefficient was 0.990 or higher.

### Total and cytosolic citrate measurement

The citrate content was determined on total or cytosolic fractions using the citrate assay kit (ab83396, Abcam) according to manufacturer’s instructions. After deproteinization using the perchloric acid, the sample was diluted with the reaction mix, and fluorescence was measured using a plate reader and the following settings: *λ*_ex_ = 535 nm; *λ*_em_ = 587 nm. Fluorescence was measured using a Versamax Tunable microplate reader (Molecular Devices) or Spark multimode microplate reader (TECAN Trading AG, Switzerland).

### EP300 activity assay

EP300 activity was determined using the SensoLyte EP300 assay kit (AS-72172, Anaspec) with some modification. Briefly, after cytosolic EP300 was immunoprecipitated from cells, the lysates were incubated with AcCoA solution and substrates (H3 or p53) for 15 min at 37 °C. Developer solution was added and incubated for 30 min at room temperature. After the reaction was stopped with stop solution, the fluorescence was measured using a plate reader (Versamax Tunable microplate reader, Molecular Devices) or Spark multimode microplate reader (TECAN Trading AG, Switzerland) and the following settings: *λ*_ex_ = 389 nm; *λ*_em_ = 513 nm.

### Co-immunoprecipitation (Co-IP)

Cells in 60 or 100 mm dishes were washed twice with PBS and lysed in ice-cold lysis buffer (40 mM HEPES (pH 7.4), 2 mM EDTA, 10 mM pyrophosphate, 10 mM glycerophosphate, and 0.3% CHAPS or 0.5% Triton X-100 and protease/phosphatase inhibitors cocktail), and further supplemented with 10 mM sodium butyrate and 1 mM TSA for IP with Ac-K antibody. Lysates were incubated on ice for 20 min and isolated by centrifugation at 16,100 g for 10 min. Supernatants were transferred to new tubes; 1/10 of the sample was kept as input control, while the remaining lysate was overnight incubated with primary antibodies at 4 °C with gentle agitation. Thereafter, Dynabeads-protein G (Life Technologies) were added to the samples and incubated at 4 °C for 2 h. Beads were washed more than three times with lysis buffer and the immunoprecipitated proteins were eluted and denatured with 2× Laemmli buffer and boiled for 10 min at 100 °C, separated by SDS-PAGE.

### GFP-Trap for IP of GFP (or YFP)-fusion proteins

The IP of GFP or YFP-fusion proteins was performed using GFP-Trap (gtma-100, ChromoTek) according to manufacturer’s instructions with some modifications. Briefly, cells in 60 mm dishes were lysed in ice-cold lysis buffer (10 mM Tris-HCl, pH 7.4, 150 mM NaCl, 0.5 mM EDTA and 0.3% CHAPS and protease/phosphatase inhibitors cocktail). 0.5 ml of cell lysate was incubated for 1 h at 4 °C with 20 µl of GFP-Trap slurry, and then the beads were washed two times with the wash buffer (10 mM Tris-HCl, pH 7.4, 150 mM NaCl, 0.5 mM EDTA). 60 µl of 2× Laemmli buffer were added, and boiled for 10 min at 100 °C, separated by SDS-PAGE.

### Image analysis

Volocity software (PerkinElmer) was used for analysis and processing of confocal images. For colocalization analysis of confocal images, we used MOC. A minimum of 30 cells were examined for each condition. All experiments were repeated at least three times (*N* = 3). The background was fixed for all within-experiment analyses. See details in the figure legends.

### Statistical analysis

Significance levels for comparisons between groups were determined with unpaired two-tailed Student’s *t* test or one-way or two-way ANOVA followed by appropriate post-hoc test for multiple comparisons using GraphPad Prism 7 (GraphPad Software) or Excel (Microsoft Office), where appropriate. For western blots, protein levels were normalized to total forms or a housekeeping protein, such as Tubulin, GAPDH or Actin. All data were expressed as means ± standard error of the mean (SEM). *p* values of <0.05 were considered statistically significant. Sample sizes were chosen on the basis of extensive experience with the assays we have performed. The experiments were appropriately randomized. We have not formally tested whether the data met the assumptions of the statistical approach, but these assays and data are typically analyzed in the literature with the approaches we have used. The statistical parameters are specified within the figure legends. Also, see Source data file for statistical source data.

### Reporting summary

Further information on research design is available in the Nature Research Reporting Summary linked to this article.

## Supplementary information

Supplementary Figures and legends

Reporting Summary

## Data Availability

The authors declare that the data supporting the findings of this study are available within the article and its Supplementary Information and Source Data. The source data underlying Figs. 1–4 and Supplementary Figs. 1–12 are provided as Source Data files. Source data are provided with this paper.

## Source data

Source data file
