# Supplementary material for: Leucine regulates autophagy via acetylation of the mTORC1 component raptor
Source: Nat Commun. 2020 Jun 19;11:3148. doi: 10.1038/s41467-020-16886-2 (PMC7305105; doi:10.1038/s41467-020-16886-2)

Supplementary Information

Leucine regulates autophagy via acetylation of the mTORC1 component raptor

Sung Min Son[1,2], So Jung Park[1], Eleanna Stamatakou[1,2], Mariella Vicinanza[1,2], Fiona M. Menzies[1] & David C. Rubinsztein[1,2*]

Supplementary Figure 1

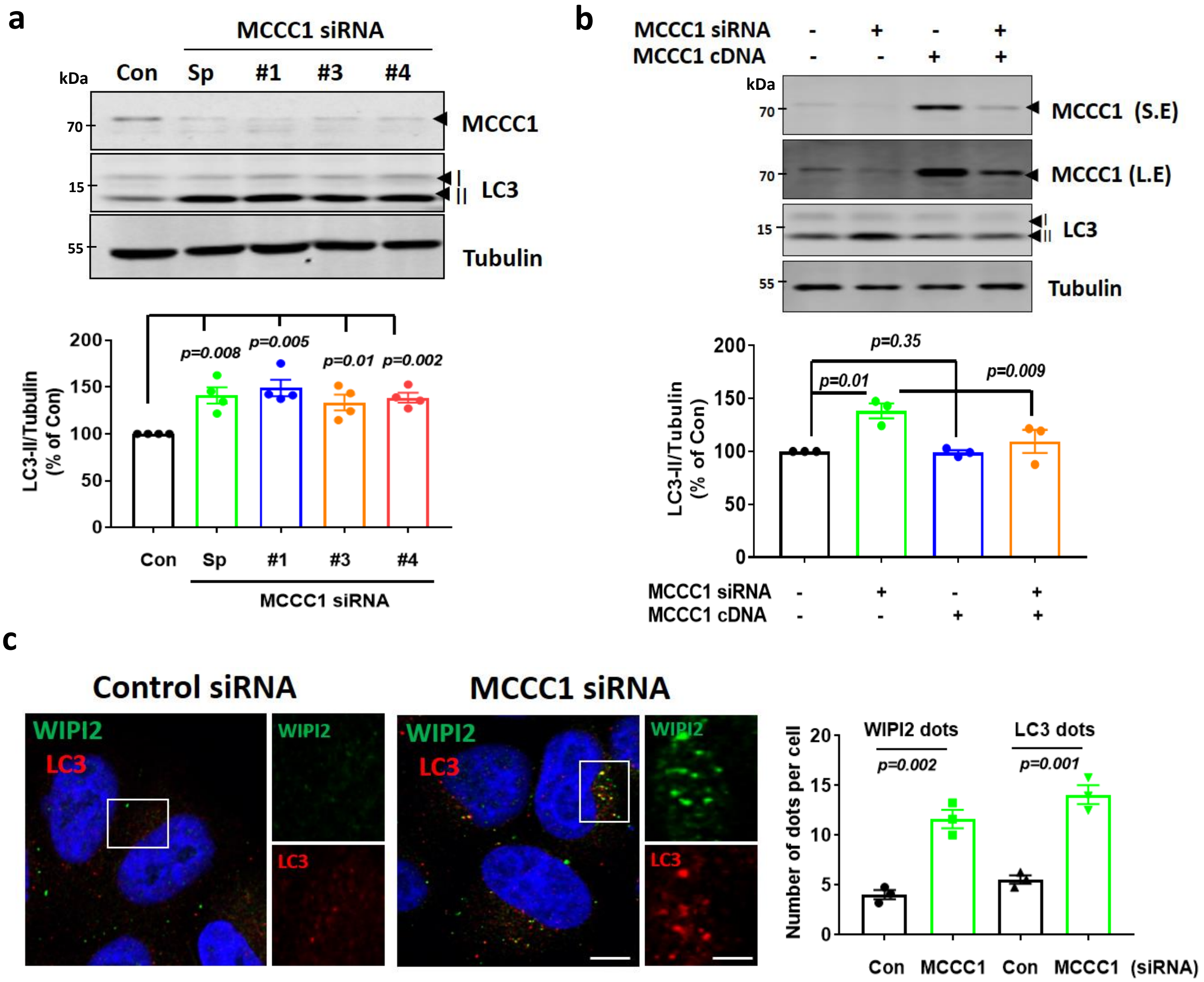

Supplementary Figure 1. MCCC1 negatively regulates autophagy in HeLa cells.

a, Representative western blot showing the effect of MCCC1 knockdown with separate deconvoluted oligonucleotides on LC3-II levels. HeLa cells were transfected with scrambled control siRNA (Con)/ MCCC1 SMARTpool (Sp)/ MCCC1 oligo #1, #3, #4 siRNA. Blots are representative of four independent experiments. (N=4) Two-tailed unpaired t-test. **b,** Re-introduction of MCCC1 cDNA into MCCC1 knockdown HeLa cells. Blots are representative of three independent experiments (N=3). Two-tailed unpaired t-test. **c,** Immunostaining of HeLa cells treated with MCCC1 SMARTpool siRNA using WIPI2 (green) and LC3 (red) antibodies, nuclei are stained with DAPI (blue). Scale bar, 5 μm, 1 μm (enlarged images). N=3, 20-25 cells scored per condition per experiment. Two-tailed unpaired t-test. Data are presented as mean values +/- SEM. Source data are provided as a Source Data file.

Supplementary Figure 2

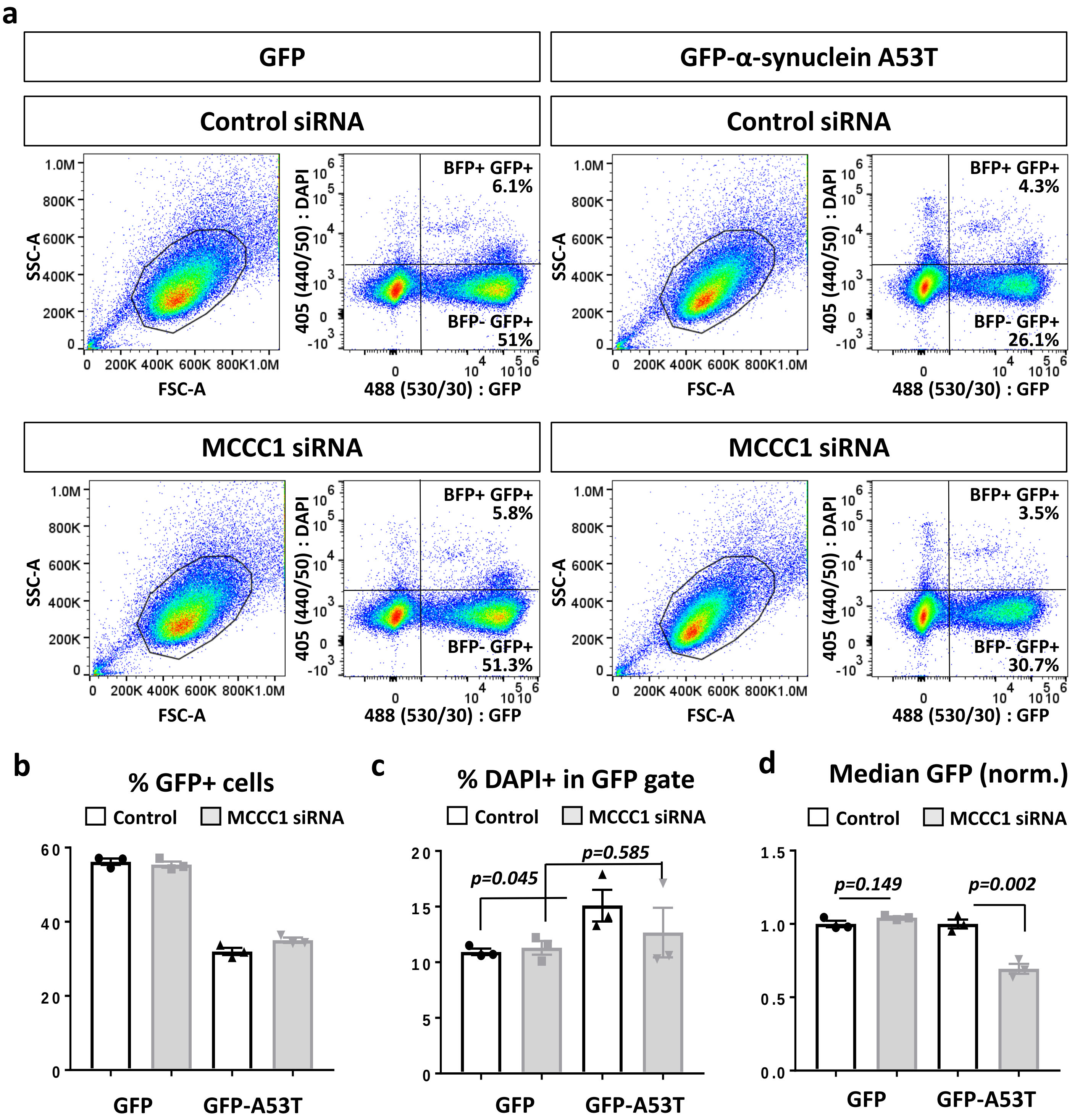

Supplementary Figure 2. Effect of MCCC1 KD on cell viability and α-synuclein A53T clearance.

Control or MCCC1 siRNA transfected HeLa cells were transfected with GFP or GFP-α-synuclein A53T. 48 h later cells were analysed using FACS after live staining with 2mg/mL DAPI. 60,000 single cells were recorded per well. **a,** No major differences were seen in FSC/SSC plots. Gating strategy: cells were first gated on forward (FSC-A) and side scatter (SSC-A) and then for singlets (FSC-A/FSC-H). 60,000 single cells were recorded. GFP+ and DAPI+ gates were set using untransfected and GFP-transfected cells not stained with DAPI. The % of GFP+ cells were unchanged between control and MCCC1 knockdown conditions **(a, b)**, suggesting that there is no effect on the cell survival of GFP- or GFP-A53T expressing cells. DAPI staining revealed a very mild effect on cell viability (BFP+GFP+ cells are dead; BFP+GFP- are alive) in control siRNA transfected cells upon GFP-A53T expression ($p=0.045$; two-tailed unpaired t-test), but not in MCCC1 siRNA transfected cells **(a, c)**, indicating that knockdown of MCCC1 rescues GFP-A53T-induced cell toxicity. GFP-A53T median fluorescence **(a, d)** is significantly decreased upon MCCC1 knockdown cells ($p=0.0026$; two-tailed unpaired t-test), confirming that knockdown of MCCC1 reduces the levels of α-synuclein A53T. Data are from 3 independently transfected wells with single mastermixes for each condition in one experiment. In this experiment we have sought to relate cell death to GFP-α-synuclein A53T fluorescence levels in response to MCCC1 knockdown. As both parameters would vary between individual experiments at different times, we considered the technical replicates as an appropriate strategy. Source data are provided as a Source Data file.

Supplementary Figure 3

a

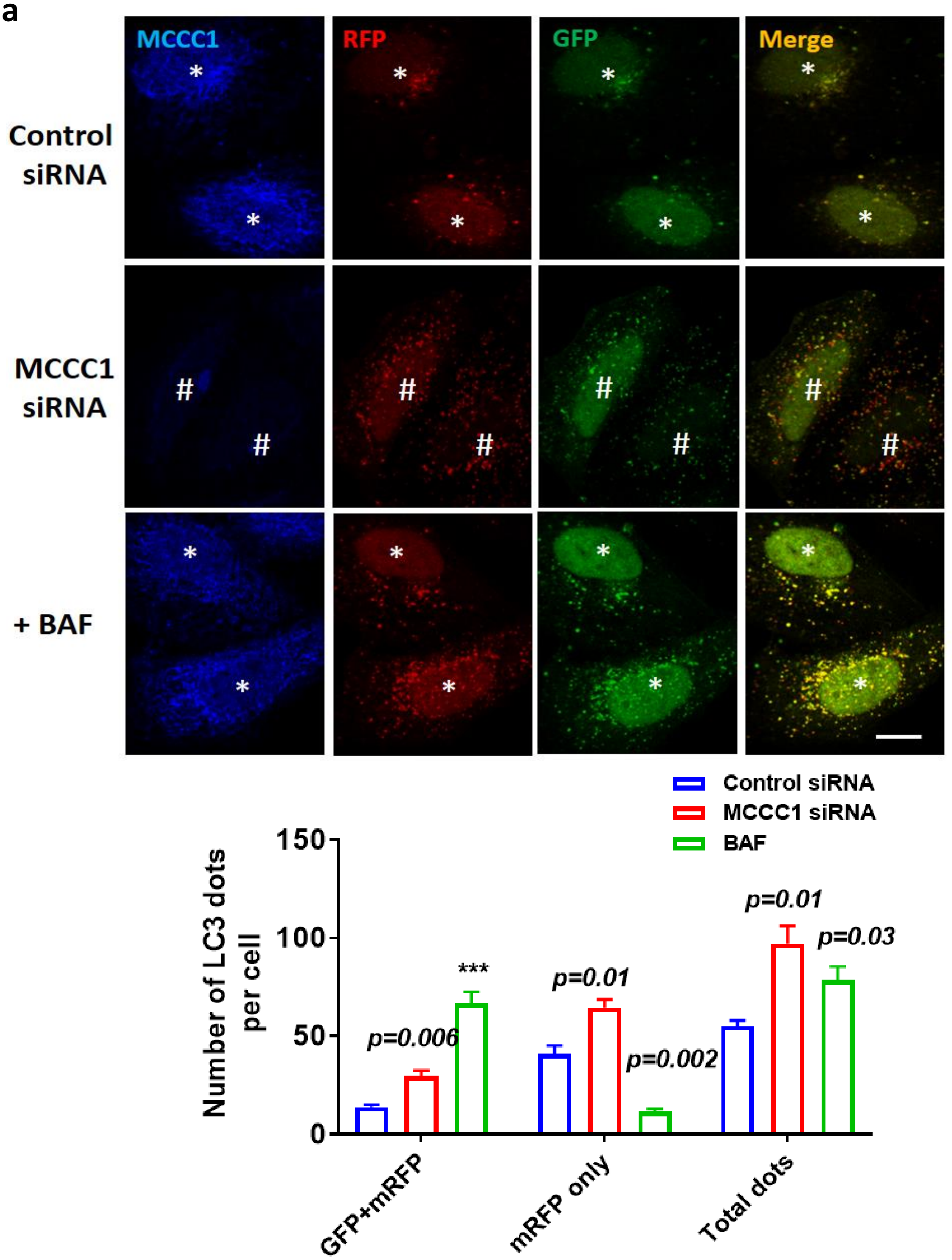

Supplementary Figure 3. Knockdown of MCCC1 activates autophagic flux in HeLa cells. a, Autophagic flux with HeLa cells stably expressing mRFP-GFP-LC3 (tfLC3). Representative confocal z-stack images (left panel) and total number of GFP/mRFP dots (autophagosomes) and mRFP-only dots (autolysosomes). Graph shows data from three biologically independent experiments (N=3; 47-70 cells analysed per condition per experiment). Scale bar, 10 μm. *** $p < 0.001$ vs. control tfLC3 expressing HeLa cells; two-tailed unpaired t-test. Data are presented as mean values +/- SEM. Source data are provided as a Source Data file.

Supplementary Figure 4

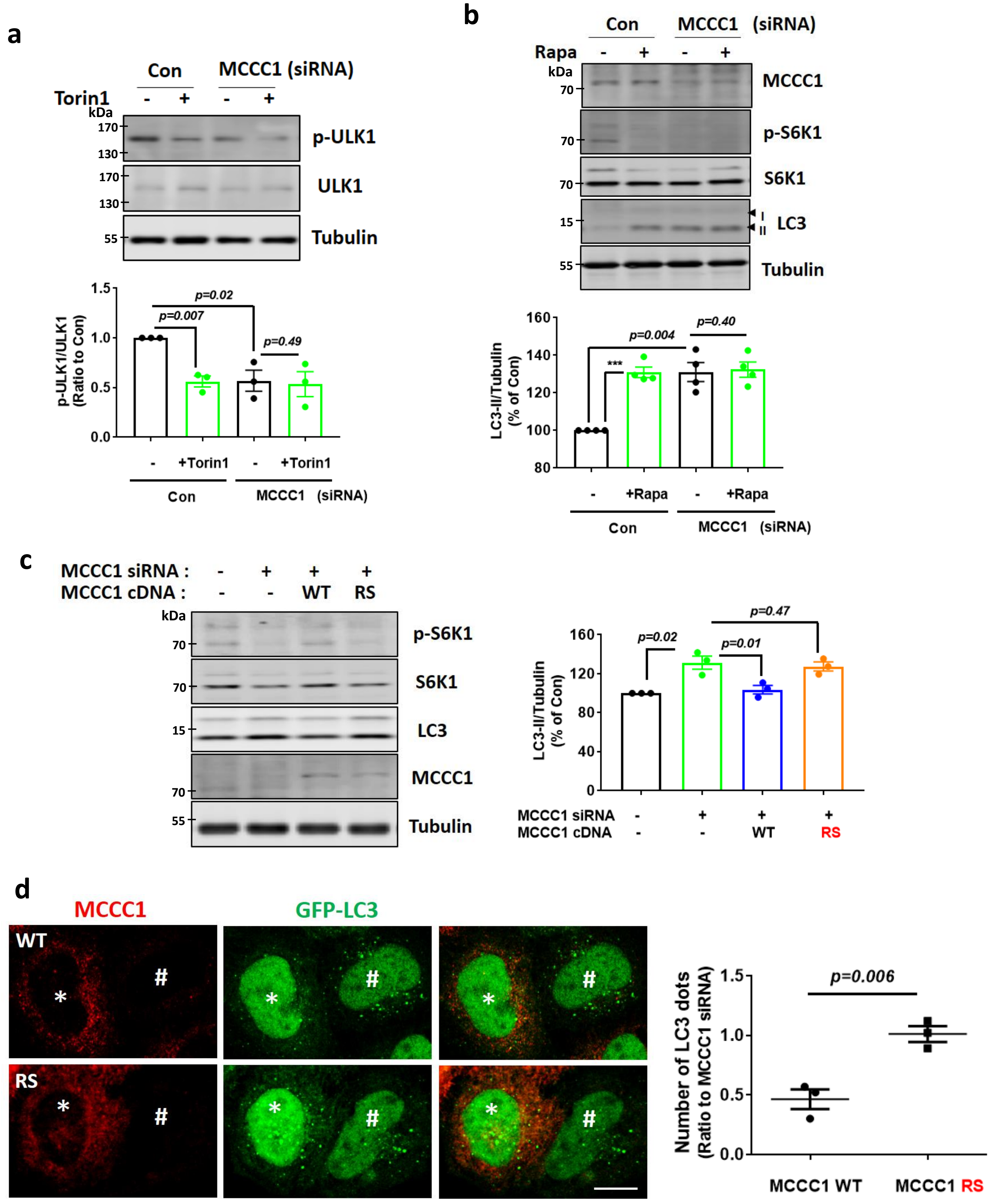

Supplementary Figure 4. MCCC1 regulates autophagy by perturbing Leu metabolism which inhibits mTORC1.

a, Effect of mTOR inhibitor Torin1 in MCCC1-knockdown HeLa cells on ULK1 phosphorylation. Control and MCCC1 knockdown cells were treated with 0.5 µM Torin1 (or DMSO) for 4 h. p-ULK1 (Ser 757). N=3. Two-tailed unpaired t-test. **b,** Effect of mTOR inhibitor rapamycin (Rapa) in MCCC1-knockdown HeLa cells. Control and MCCC1 knockdown cells were treated with 0.5 µM Rapa (or DMSO) for 24 h. N=4. *** $p < 0.001$ vs. control cells; two-tailed unpaired t-test. **c,** Increased autophagy resulting from defect in Leu catabolism using MCCC1 WT or RS mutant in MCCC1-knockdown HeLa cells. Cells were depleted of MCCC1 with siRNA and reconstituted with MCCC1 WT or RS mutant, then analysed for phosphorylated S6K1 and LC3-II levels in HeLa cells. N=3. Two-tailed unpaired t-test. **d,** Increased autophagy levels in MCCC1 mutant-expressing cells. GFP-LC3 overexpressing HeLa cells were depleted of MCCC1 with siRNA and transfected with cDNA constructs encoding either MCCC1 WT or the inactive MCCC1 RS mutant. # MCCC1 knockdown cells, * MCCC1 expressing cells. Cells were immunostained for MCCC1 (red) then analysed for the number of GFP positive punctae. N=3, 30-40 cells analysed per condition per experiment. Two-tailed unpaired t-test. Scale bar, 10 µm. Data are presented as mean values +/- SEM. Source data are provided as a Source Data file.

Supplementary Figure 5

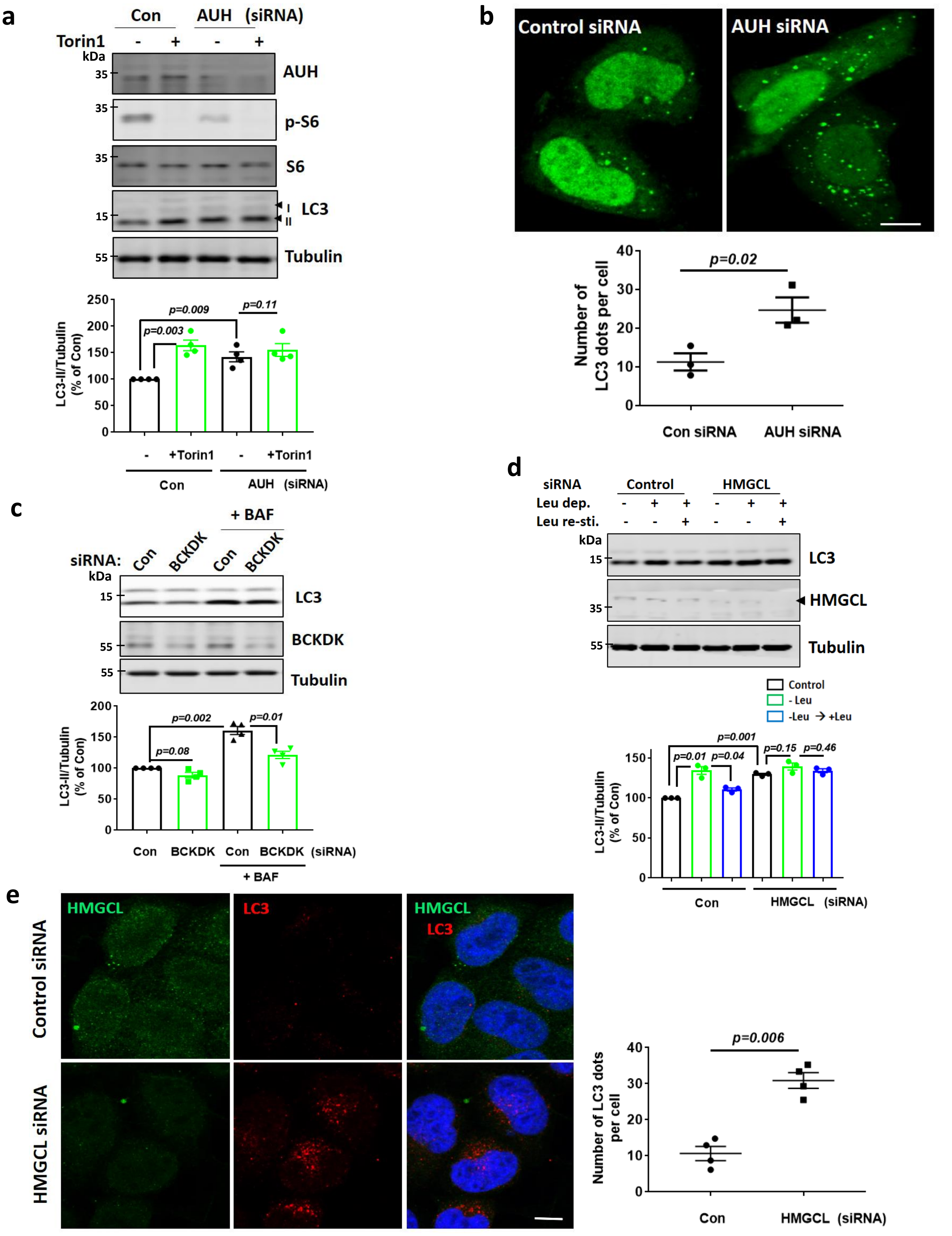

Supplementary Figure 5. Other Leu catabolic enzymes also regulates autophagy through control of Leu metabolism.

a, Effect of Torin1 in AUH-knockdown HeLa cells. Control and AUH knockdown cells were treated with 0.5 µM Torin1 (or DMSO) for 4 h. Two-tailed unpaired t-test. p-S6 (Ser235/236). N=4. **b,** Increased LC3 dot numbers in AUH-knockdown HeLa cells. N=3, 40-50 cells analysed per condition per experiment. Two-tailed unpaired t-test. Scale bar, 10 µm. **c,** Decreased LC3-II levels in BCKDK-knockdown HeLa cells. N=4. Two-tailed unpaired t-test. **d,** Autophagy regulation by Leu in HMGCL-knockdown HeLa cells. HeLa cells were treated with scrambled siRNA or HMGCL siRNA then incubated in Leu-depleted media for 4 h or incubated in Leu-depleted media for 4 h, followed by the re-addition of 10 µM of Leu to the media for 1 h. N=3. Two-tailed unpaired t-test. **e,** Immunostaining of HeLa cells treated with HMGCL siRNA using HMGCL (green) and LC3 (red) antibodies, nuclei are stained with DAPI (blue). Scale bar, 5 µm. N=4. 40-50 cells analysed per condition per experiment. Two-tailed unpaired t-test. Data are presented as mean values +/- SEM. Source data are provided as a Source Data file.

Supplementary Figure 6

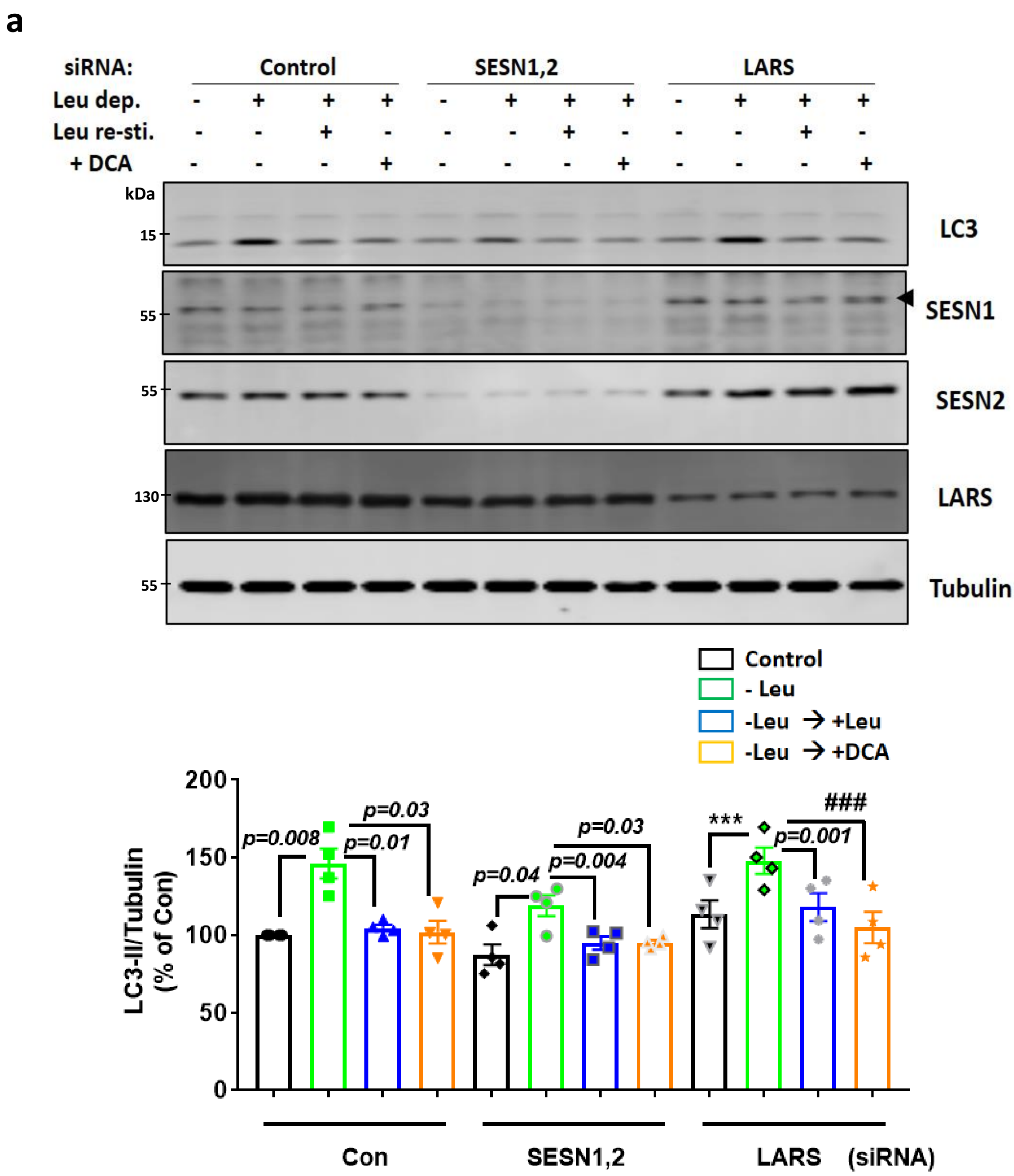

Supplementary Figure 6. Leu regulates autophagy in HeLa cells via a downstream metabolite.

a, Autophagy regulation by Leu or DCA in SESN1, 2- or LARS-knockdown HeLa cells. HeLa cells were treated with scrambled siRNA or SESN1, 2- or LARS siRNA then incubated in Leu-depleted media for 4 h or incubated in Leu-depleted media for 4 h, followed by the re-addition of 10 μM of Leu or 10 mM DCA to the media for 1 h. Blots are representative of four independent experiments (N=4). *** $p < 0.001$ vs. control cells; ### $p < 0.001$ vs. Leu-depleted cells; two-way ANOVA with post hoc Tukey's multiple comparison test. Data are presented a s mean values +/- SEM. Source data are provided as a Source Data file.

Supplementary Figure 7

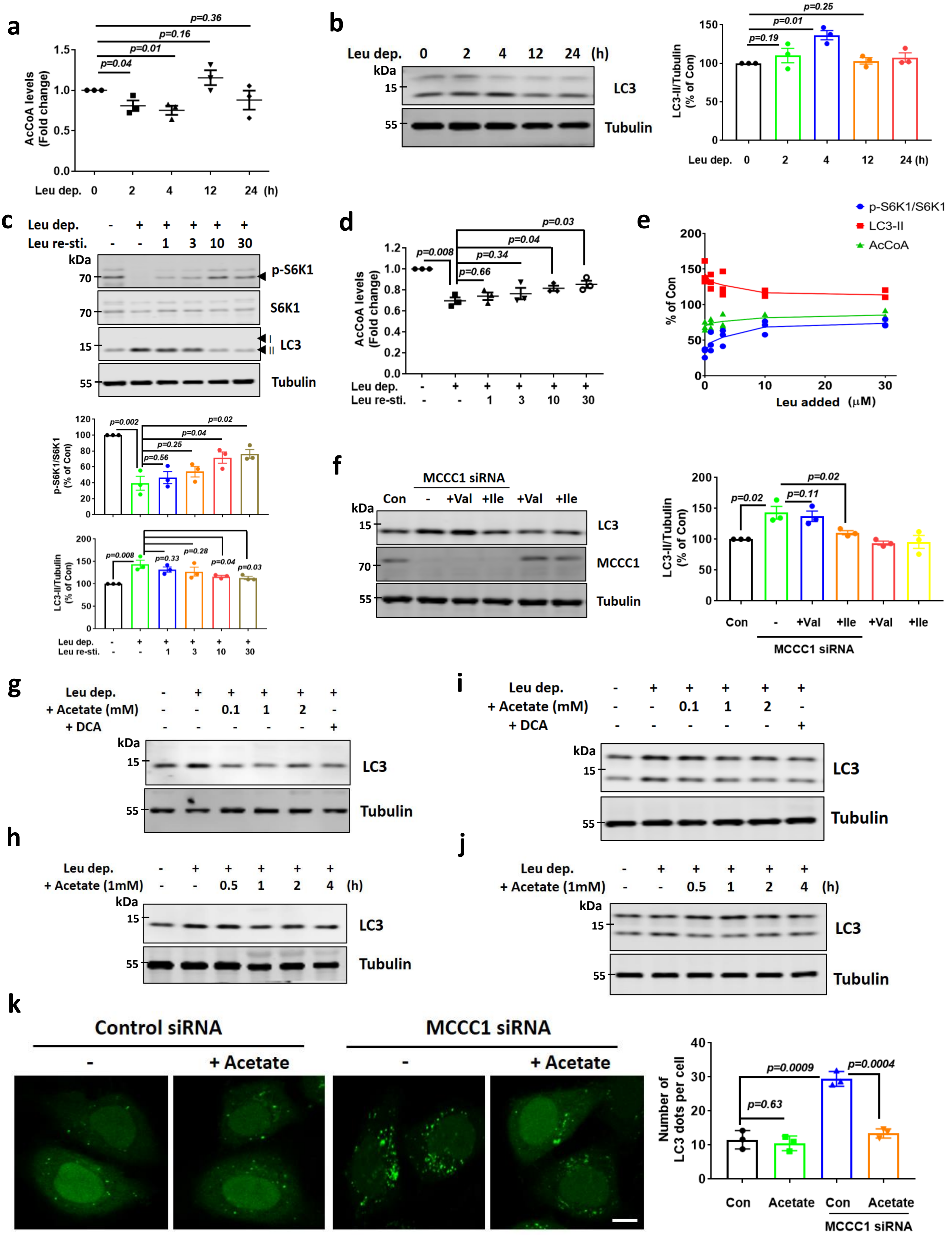

Supplementary Figure 7. AcCoA is essential for Leu metabolism-mediated autophagy regulation.

a, Effect of incubation time (0, 2, 4, 12, 24 h) in Leu-depleted media on AcCoA levels in HeLa cells. Graph shows data from four independent biological replicates (N=4); two-tailed unpaired t-test. **b,** Effect of incubation time (0, 2, 4, 12, 24 h) in Leu-depleted media on LC3-II levels. Blots are representative of three independent experiments (N=3); two-tailed unpaired t-test. **c,** Rescue of mTORC1 and autophagy by adding back Leu in Leu-depleted media in HeLa cells. Cells were incubated in Leu-depleted media for 4 h or incubated in Leu-depleted media for 4 h, followed by the re-addition of indicated concentration of Leu to the media for 1 h. N=3. Unit, µM. Two-tailed unpaired t-test. **d,** Rescue of reduced AcCoA levels by adding back Leu in Leu-depleted HeLa cells; N=3; two-tailed unpaired t-test. **e,** The EC50 values in this graph are: p-S6K1/S6K1 13.077 ± 7.752 / LC3-II 8.4 ± 0.794 / AcCoA 26.667 ± 11.93 (standard deviations; SD). Note that these EC50 values are not simple data as one would get for receptor binding as a function of drug concentration. The output in all of these processes that have been assayed is really a flux rather than a static value. For example, some of the AcCoA produced from Leu is used to acetylate various proteins, and some is consumed in other cellular reactions that involve AcCoA. Thus, the AcCoA that has been measured is an underestimated of the AcCoA that was produced by this pathway (and the same caveat applies to some extent for the other parameters). Nevertheless, the EC50 values for p-S6K1/S6K1, LC3-II and AcCoA are in a similar range with overlapping standard deviations. **f,** Autophagy regulation by other branched chain amino acids (BCAAs; isoleucine (Ile) and valine (Val)) in MCCC1-knockdown HeLa cells. 0.1 mM Ile, capable of generating AcCoA, rescued autophagy activation by MCCC1 depletion. N=3; two-tailed unpaired t-test. **g, h,** Autophagy regulation by treatment of sodium acetate with different concentration (g) or time (h) to Leu-depleted HeLa cells. 10 mM of DCA was used. N=3. **i, j,** Autophagy regulation by sodium acetate with different concentrations (i) or time (j) in Leu-depleted SH-SY5Y cells. N=3. **k,** Rescue of activated autophagy in MCCC1 depleted cells by 1 mM sodium acetate. Acetate treatment restored the increased LC3 dots in MCCC1-depleted cells. N=3, 20 cells analysed per condition per experiment. Two-tailed unpaired t-test. Scale bar, 5 µm. Data are presented as mean values +/- SEM. Source data are provided as a Source Data file.

Supplementary Figure 8

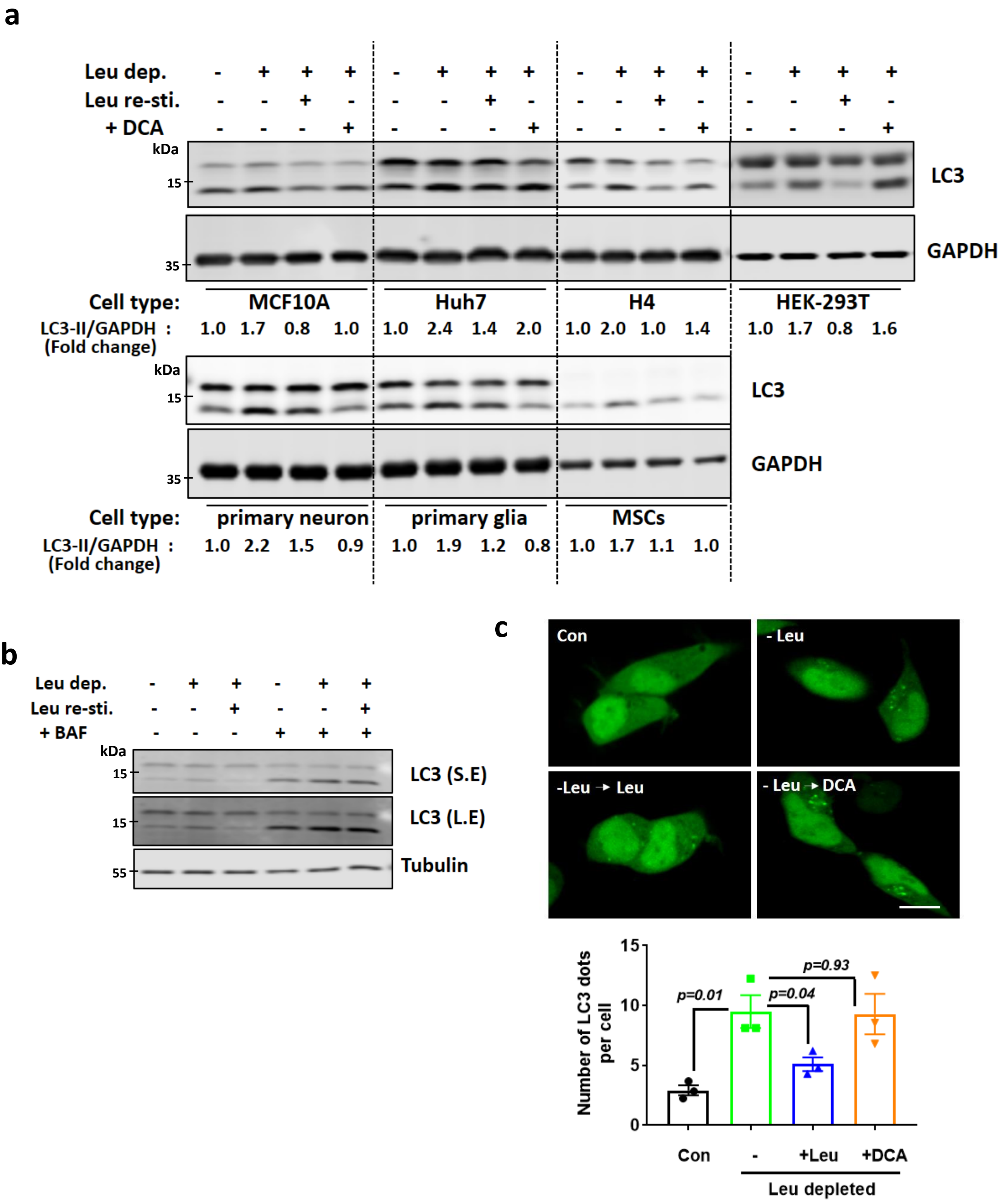

Supplementary Figure 8. AcCoA is essential for Leu metabolism-mediated autophagy regulation in most, but not all, cell types.

a, Autophagy regulation by Leu or DCA under Leu-depleted conditions in several cell types. Cells were incubated in Leu depleted media for 4 h or in Leu depleted media for 4 h with the addition of 1-10 mM DCA or 10 µM Leu for 1 h. Cells were lysed and western blots for LC3 are shown. LC3-II levels were quantified relative to GAPDH as a loading control. MSCs, mesenchymal stem cells. Blots are representative of three independent experiments. (N=3) **b,** Rescue of LC3-II levels by Leu in Leu-depleted HEK-293T cells. HeLa cells were treated with 400nM BAF for 4 h. N=4. **c,** The number of LC3 dots under Leu depletion with or without 10 µM Leu or 10 mM DCA on HEK-293T cells. N=3, 25 cells analysed per condition per experiment. Two-tailed unpaired t-test. Scale bar, 5 µm. Data are presented as mean values +/- SEM. Source data are provided as a Source Data file.

Supplementary Figure 9

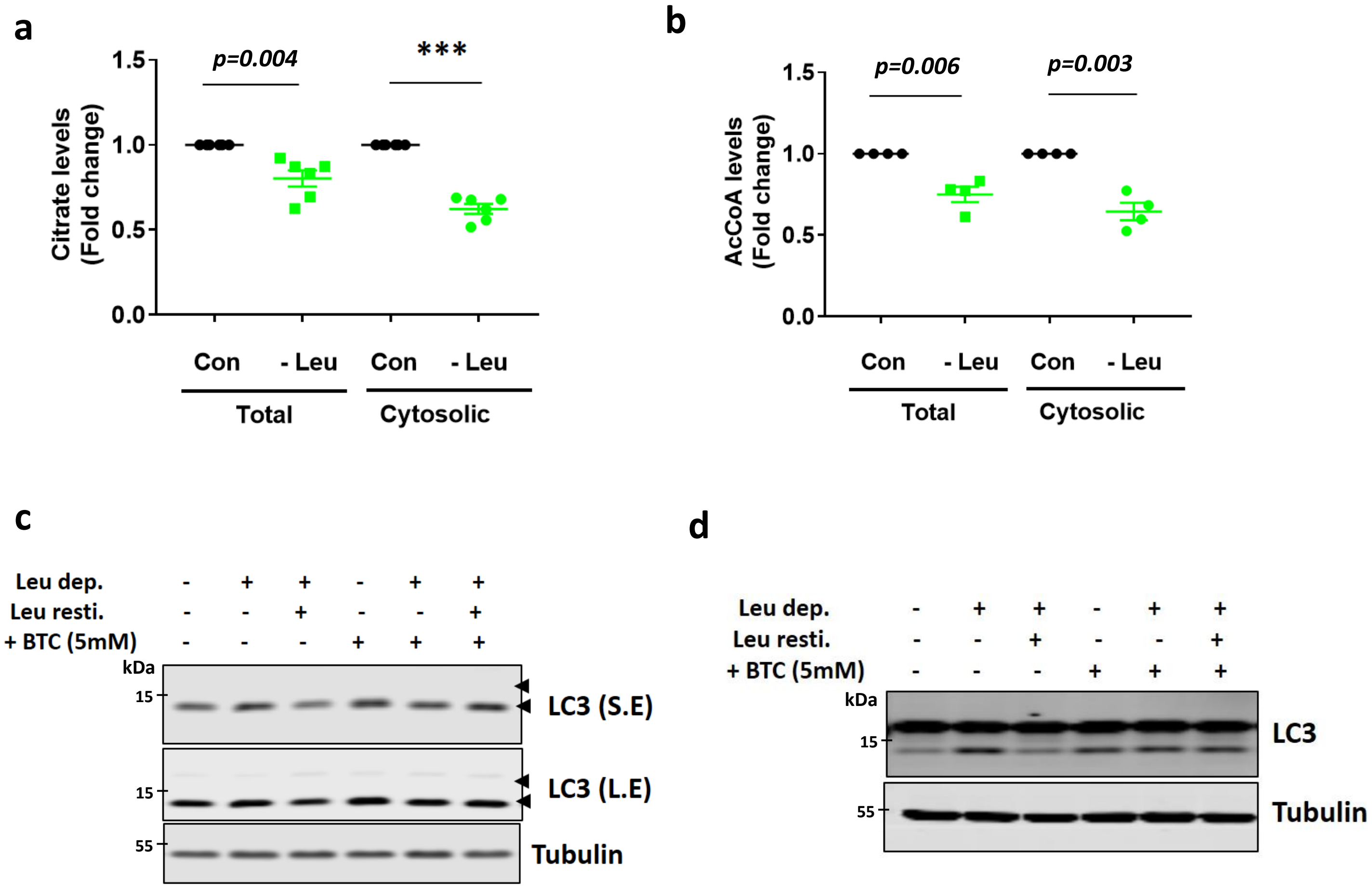

Supplementary Figure 9. Leu deprivation activates autophagy by regulating cytosolic AcCoA levels.

a, b, Total and cytosolic citrate (**a**) or AcCoA levels (**b**) were assessed using the assay kit. HeLa cells were incubated Leu-depleted conditions for 4 h followed by lysis and measurement of AcCoA levels. N=6 for citrate, N=4 for AcCoA. *** $p < 0.001$ vs. HeLa cells incubated in complete culture media; two-tailed unpaired t-test. **c, d,** Increased LC3-II level and failure of rescue by Leu in BTC-treated HeLa (**c**) or SH-SY5Y cells (**d**). 5 mM BTC was treated to cells for 4 h. Blots are representative of four independent experiments. (N=4) For LC3-II western blots, both long exposure (L.E.) and short exposure (S.E.) of the membrane to film are shown to allow for conditions under which weaker signals can be seen and stronger signals are not over-exposed. Data are presented as mean values +/- SEM. Source data are provided as a Source Data file.

Supplementary Figure 10

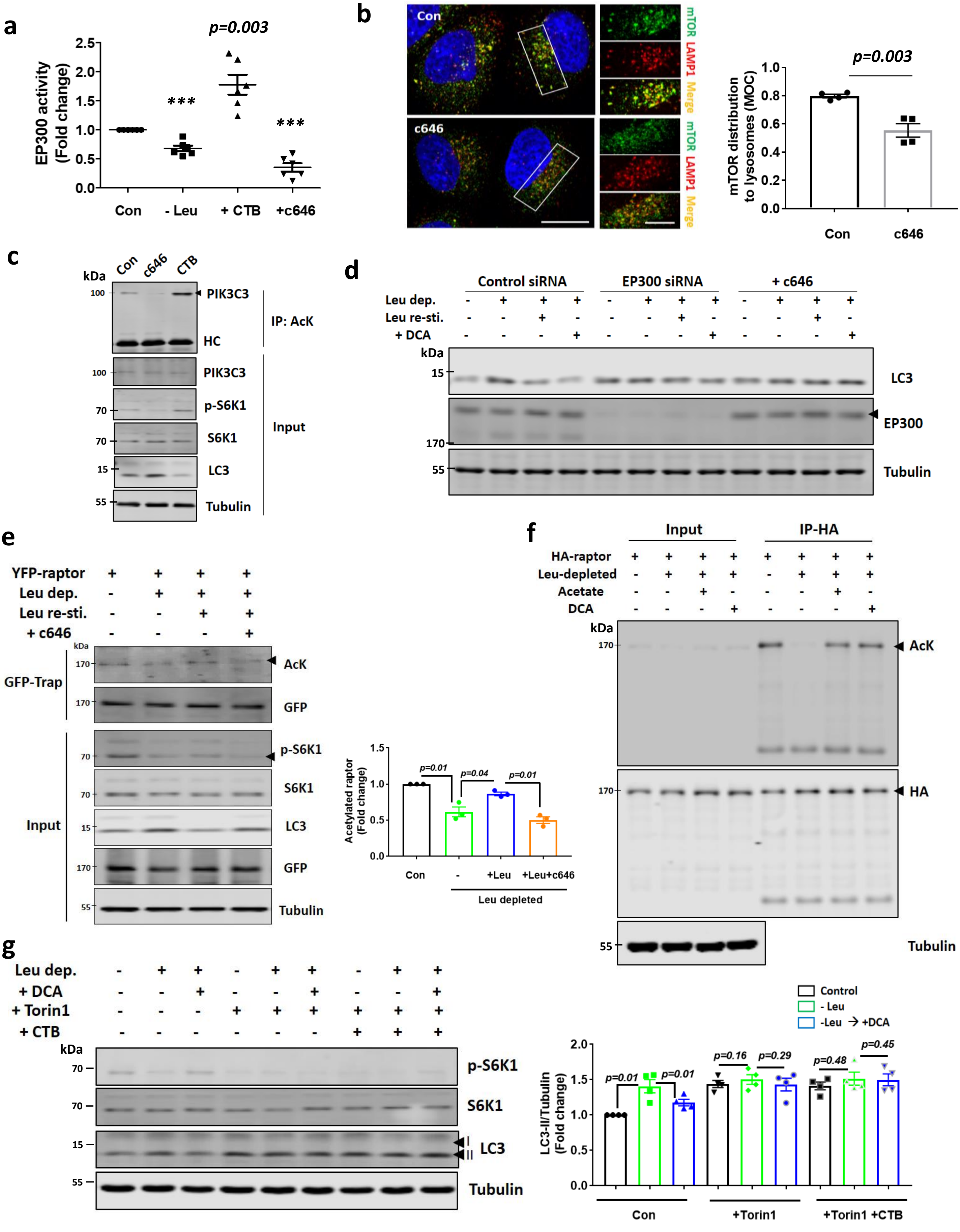

Supplementary Figure 10. EP300 inactivation-induced raptor deacetylation mediates autophagy activation after Leu deprivation.

a, Decreased EP300 activity in Leu-depleted HeLa cells. CTB, EP300 activator; c646, EP300 inhibitor. Graph shows data from six independent experiments (N=6). *** $p < 0.001$ vs. control cells; two-tailed unpaired t-test. **b,** Cells were either left untreated or c646 treated for 4 h, and then immunostained with mTOR (green) and LAMP1 (red) antibodies as shown. Co-localization panels show an overlap between mTOR and LAMP1 signals. The fraction of mTOR-positive lysosomes were determined using Volocity software. N=4, 30 cells analysed per condition per experiment. Two-tailed unpaired t-test. Scale bar, 10 μm, 2 μm (enlarged images). **c,** mTORC1 and autophagy regulation by EP300 activity using EP300 activator CTB and inhibitor c646. Acetylated PIK3C3 is decreased by c646, and is increased by CTB treatment. N=3. **d,** Increased LC3-II level and failure of rescue by Leu or DCA in EP300-knockdown or EP300 inhibitor-treated HeLa cells. HeLa cells were treated with scrambled siRNA or EP300 siRNA then incubated in Leu-depleted media for 4 h or incubated in Leu-depleted media for 4 h, followed by the re-addition of 10 μM of Leu or 10 mM DCA to the media for 1 h. N=3. **e,** Decreased acetylation of raptor by Leu depletion. N=3. One-way ANOVA with post hoc Tukey's multiple comparison test. **f,** Regulation of raptor acetylation by sodium acetate and DCA. Cells were incubated in Leu-depleted media for 4 h or incubated in Leu-depleted media for 4 h, followed by the re-addition of 1 mM of sodium acetate or 10 mM DCA to the media for 1 h. N=3. **g,** The autophagy regulation on Torin1-treated HeLa cells w/ or w/o CTB. Cells were treated with 50 μM CTB for 12 h w/ or w/o 0.5 mM Torin1 for the last 4 h. N=4. Two-way ANOVA with post hoc Tukey's multiple comparison test. Data are presented as mean values +/- SEM. Source data are provided as a Source Data file.

Supplementary Figure 11

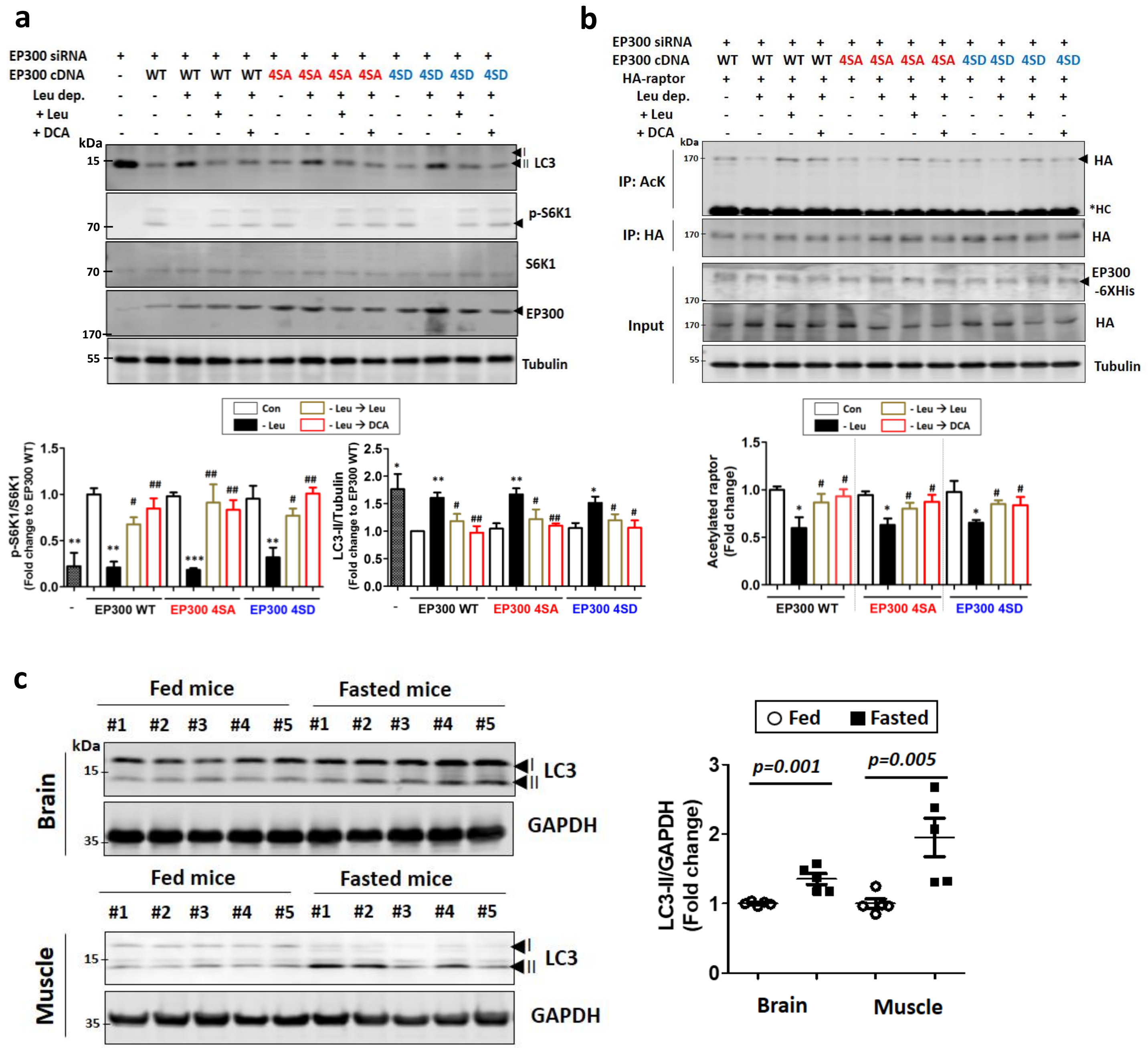

Supplementary Figure 11. mTORC1 activity, as downstream signal of EP300, is a critical regulator for autophagy by AcCoA under Leu-depleted conditions.

a, mTORC1 and autophagy regulation in EP300 WT-, 4SA- or 4SD-mutant expressing HeLa cells. Cells were depleted of endogenous EP300 with siRNA and reconstituted with 6XHis tagged EP300 WT, 4SA or 4SD cDNA, then incubated in Leu-depleted media for 4 h or incubated in Leu-depleted media for 4 h, followed by the re-addition of 10 µM of Leu or 10 mM DCA to the media for 1 h. Graph shows data from three biologically independent experiments (N=3). * $p < 0.05$, ** $p < 0.01$ vs. control HeLa cells; # $p < 0.05$, ## $p < 0.01$ vs. Leu-depleted cells; two-way ANOVA with post hoc Tukey's multiple comparison test. **b,** Acetylation of raptor in EP300 WT-, 4SA- or 4SD-mutant expressing HeLa cells. Graph shows data from three biologically independent experiments (N=3). * $p < 0.05$ vs. control HeLa cells; # $p < 0.05$ vs. Leu-depleted cells; two-way ANOVA with post hoc Tukey's multiple comparison test. **c,** Increased LC3-II levels in fasted mice brains and livers. After 22.5 h starvation, mice were given free access to food for 1.5 h followed by a second round of starvation for another 22.5 h. The tissue samples from fed (n = 5) and fasted mice (n = 5) were analyzed for LC3-II levels. Two-tailed unpaired t-test. Data are presented as mean values +/- SEM. Source data are provided as a Source Data file.

Supplementary Figure 12

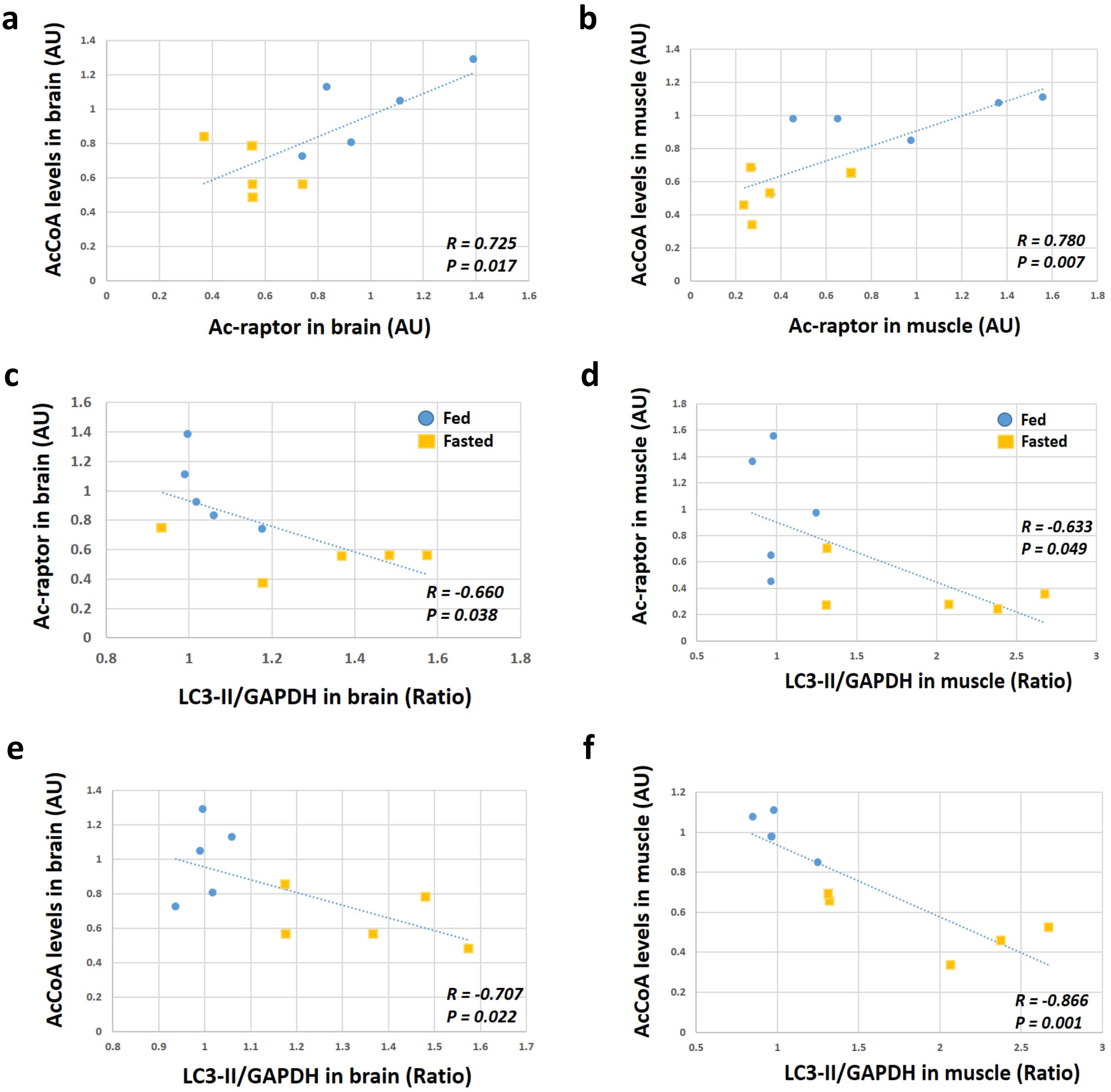

Supplementary Figure 12. Correlations between acetylated raptor and AcCoA levels with autophagy in brains and muscles of fed and fasted mice.

a, b, Correlation between acetylated raptor and AcCoA levels in fed and fasted mice brains (a) or muscles (b). **c, d,** Correlation between acetylated raptor and LC3-II levels in fed and fasted mice brains (c) or muscles (d). **e, f,** Correlation between AcCoA levels and LC3-II levels in fed and fasted mice brains (e) or muscles (f). Two-tailed t-test. R score is Pearson's *r* correlation coefficient. Source data are provided as a Source Data file.

Supplementary Figure 13. Full scans of uncropped blots.

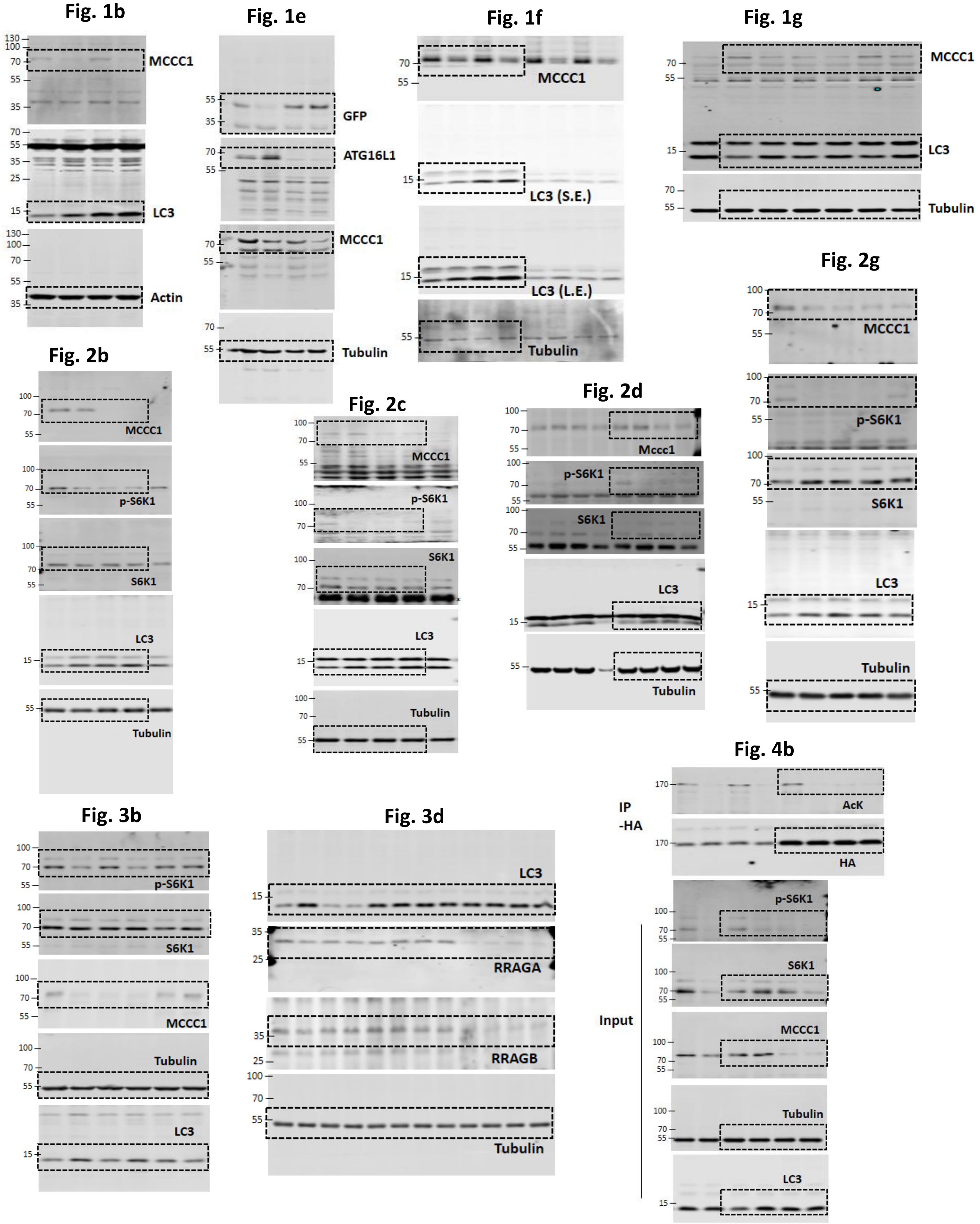

Supplementary Figure 13. Continued Full scans of uncropped blots.

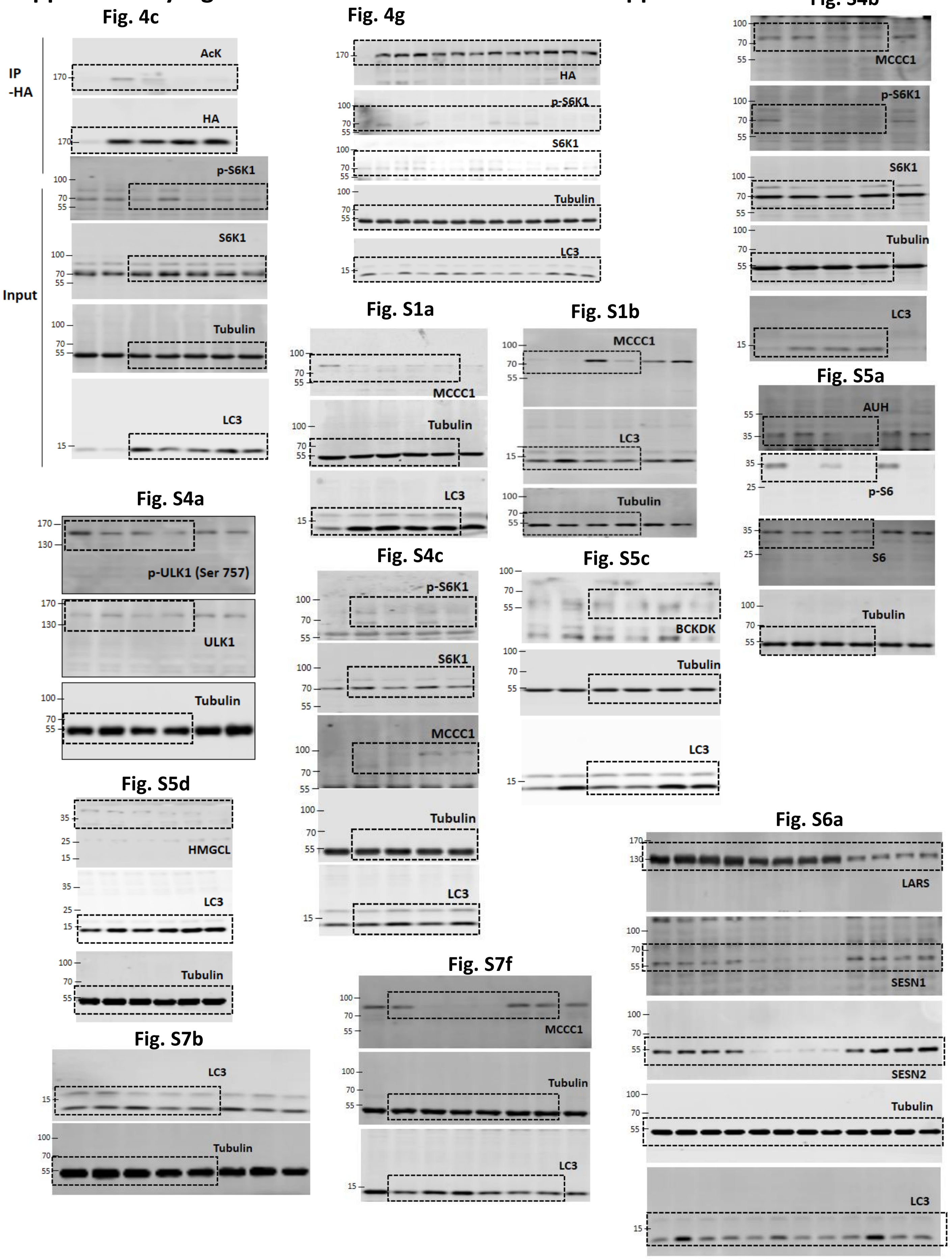

Supplementary Figure 13. Continued Full scans of uncropped blots.

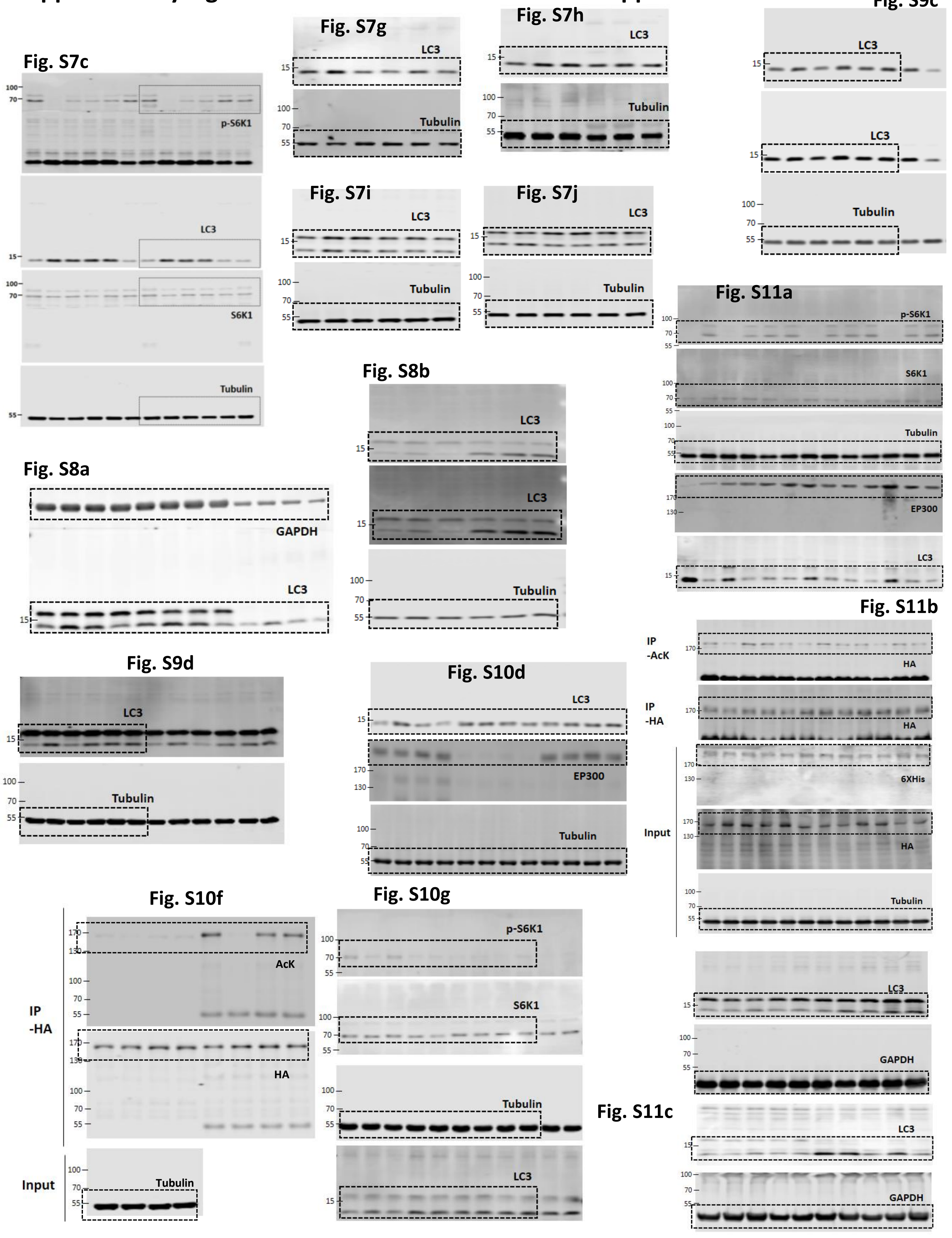

Supplement: Supplementary file 1 — Supplementary Figures and legends [file 41467_2020_16886_MOESM1_ESM.pdf]
