# Supplementary material for: Leucine regulates autophagy via acetylation of the mTORC1 component raptor
Source: Nat Commun. 2020 Jun 19;11:3148. doi: 10.1038/s41467-020-16886-2 (PMC7305105; doi:10.1038/s41467-020-16886-2)
Supplement: Supplementary file 2 — Reporting Summary [file 41467_2020_16886_MOESM2_ESM.pdf]

nature research

Corresponding author(s): Prof. David C Rubinsztein

Last updated by author(s): May 12, 2020

Reporting Summary

Nature Research wishes to improve the reproducibility of the work that we publish. This form provides structure for consistency and transparency in reporting. For further information on Nature Research policies, see Authors & Referees and the Editorial Policy Checklist.

Statistics

For all statistical analyses, confirm that the following items are present in the figure legend, table legend, main text, or Methods section.

n/a	Confirmed	
☐	☒	The exact sample size (*n*) for each experimental group/condition, given as a discrete number and unit of measurement
☐	☒	A statement on whether measurements were taken from distinct samples or whether the same sample was measured repeatedly
☐	☒	The statistical test(s) used AND whether they are one- or two-sided<br>*Only common tests should be described solely by name; describe more complex techniques in the Methods section.*
☐	☒	A description of all covariates tested
☐	☒	A description of any assumptions or corrections, such as tests of normality and adjustment for multiple comparisons
☐	☒	A full description of the statistical parameters including central tendency (e.g. means) or other basic estimates (e.g. regression coefficient) AND variation (e.g. standard deviation) or associated estimates of uncertainty (e.g. confidence intervals)
☐	☒	For null hypothesis testing, the test statistic (e.g. *F*, *t*, *r*) with confidence intervals, effect sizes, degrees of freedom and *P* value noted<br>*Give P values as exact values whenever suitable.*
☒	☐	For Bayesian analysis, information on the choice of priors and Markov chain Monte Carlo settings
☒	☐	For hierarchical and complex designs, identification of the appropriate level for tests and full reporting of outcomes
☐	☒	Estimates of effect sizes (e.g. Cohen's *d*, Pearson's *r*), indicating how they were calculated

Our web collection on statistics for biologists contains articles on many of the points above.

Software and code

Policy information about availability of computer code

Data collection	Gels were imaged using LICOR-Odyssey apparatus using IMAGE STUDIO Lite Licor version 5.2<br>Confocal Carl Zeiss LSM710, LSM780 and LSM880 operated with ZEN imaging software version 2.6<br>Microscope imaging system (Nikon Eclipse E600)<br>Attune NxT Flow Cytometer (ThermoFisher Scientific)<br>a Versamax Tunable microplate reader (Molecular Devices) or Spark multimode microplate reader (TECAN Trading AG, Switzerland)
Data analysis	IMAGE STUDIO Lite Licor version 5.2 and Image J version 1.52p for gel analysis.<br>ZEN imaging software version 2.6 for microscopic image analysis.<br>Volocity Software version 6.2 (PerkinElmer) for Mander's Overlap Coefficient (MOC) or Pearson's correlation coefficient (PCC).<br>Microsoft Excel 2016 and GraphPad Prism 7.0 (GraphPad Software) for statistical analysis.<br>FlowJo v10 software for analysis of Flow cytometry data

For manuscripts utilizing custom algorithms or software that are central to the research but not yet described in published literature, software must be made available to editors/reviewers. We strongly encourage code deposition in a community repository (e.g. GitHub). See the Nature Research guidelines for submitting code & software for further information.

Data

Policy information about availability of data

All manuscripts must include a data availability statement. This statement should provide the following information, where applicable:

- Accession codes, unique identifiers, or web links for publicly available datasets
- A list of figures that have associated raw data
- A description of any restrictions on data availability

The authors declare that the data supporting the findings of this study are available within the article and its Supplementary Information and Source Data. Source data are provided as a Source Data file.

Field-specific reporting

Please select the one below that is the best fit for your research. If you are not sure, read the appropriate sections before making your selection.

☒ Life sciences ☐ Behavioural & social sciences ☐ Ecological, evolutionary & environmental sciences

For a reference copy of the document with all sections, see nature.com/documents/nr-reporting-summary-flat.pdf

Life sciences study design

All studies must disclose on these points even when the disclosure is negative.

Sample size	Sample sizes were chosen on the basis of extensive experience with the assays we have performed. We followed the conventional way of quantification accepted in many of the published paper in the research field and determined the sample size. Sample sizes are clearly stated in the figure legends and methods section.
Data exclusions	No data were excluded from the analysis.
Replication	All experiments were repeated by three or four times. We used the average of each triplicate as a biological replicate for statistical analyses. Indicated in the figure legends, biological duplicates/triplicates were performed with similar results.
Randomization	We did not exclude any samples from the analysis, and all the experiments and tests were randomly assigned. For cell-based experiments, cell lines and primary cells were divided equally to each group and then treated with drug agents. For animal studies, mice were randomly distributed into group (n=6 each groups).
Blinding	The experimenters were blinded to the slide names for quantification of autophagosomes, autolysosomes or polyQ aggregates. During other experiments and outcome assessment, the experimenters were not blinded to allocation because treatments with different drugs made it difficult to blind and all experimentation were performed by the experimenters. Analysis using softwares minimized the occurrence of any bias.

Reporting for specific materials, systems and methods

We require information from authors about some types of materials, experimental systems and methods used in many studies. Here, indicate whether each material, system or method listed is relevant to your study. If you are not sure if a list item applies to your research, read the appropriate section before selecting a response.

Materials & experimental systems

n/a	Involved in the study	
☐	☒	Antibodies
☐	☒	Eukaryotic cell lines
☒	☐	Palaeontology
☐	☒	Animals and other organisms
☒	☐	Human research participants
☒	☐	Clinical data

Methods

n/a	Involved in the study	
☒	☐	ChIP-seq
☐	☒	Flow cytometry
☒	☐	MRI-based neuroimaging

Antibodies

Antibodies used	The following antibodies have been used in this work: mouse anti-Flag M2 (#F3165), rabbit anti-Actin (#A2066) and mouse anti-α-Tubulin (#T9026) from Sigma Aldrich; mouse anti-GAPDH clone 6C5 (#ab8245), rabbit anti-MCCC1 (#ab178675), rabbit anti-SESN1 (#ab134091), rabbit anti-LC3B (#ab192890), rabbit anti-HMGCL (#ab97293), mouse anti-WIPI2 (#ab105459) and rabbit anti-AUH (#ab157453) from Abcam; mouse anti-BCKDK (#NBP1-47664), goat anti-HA (#NB600-362) and rabbit anti-MCCC1 (#NBP1-81254) from Novus Biologicals; rabbit anti-EP300 (#sc-585), goat polyclonal anti-raptor (#sc-27744), mouse anti-HMGCL (#sc-100548), and mouse anti-MCCC1 (#sc-365754) from SantaCruz Biotechnology; mouse anti-LC3B (#0231-100; Nanotools); rabbit anti-ATG16L1 (#PM040; MBL);mouse anti-GFP (#632375 and #632592; Clontech); mouse anti-HA.11 clone 16B12 (#MMS-101P, Covance); mouse anti-EP300 (#05-257) from Millipore; mouse anti-LAMP1 clone H4A3 (obtained from Developmental Studies Hybridoma Bank, University of Iowa); rabbit anti-LAMP1 (#9091), rabbit anti-mTOR (#2972), rabbit anti-phospho-mTOR (Ser2481; #2972), rabbit anti-raptor (#2280), rabbit anti-phospho- p-S6K1 (Thr389; #9234), anti-total S6K1 (#9202), rabbit anti-phospho-S6 Ribosomal Protein (p-S6) (Ser235/236; #4856), rabbit anti-S6 Ribosomal Protein (S6) (#2217), rabbit anti-phospho-4E-BP1 (Thr37/46; #9459), rabbit anti-4E-BP1 (#9452), rabbit anti-phospho-ULK1 (Ser757; #6888), rabbit anti-ULK1 (#4773), rabbit anti-SESN2 (#8487), rabbit anti-LARS (#13868), rabbit anti-PIK3C3 (#4263), rabbit anti-acetylated-Lysine (Ac-K) (#9814, #9441), rabbit anti- RRAGA (#4357), rabbit anti- RRAGB (#8150), rabbit anti-WIPI2 (#8567), and rabbit anti-ATG16L1 (#8089) from Cell Signaling Technology; anti-mouse (#NA931V) and anti-rabbit (#NA934V) horseradish peroxidise (HRP)-conjugated secondary antibodies (GE Healthcare); anti-goat horseradish peroxidise (HRP)-conjugated secondary antibody (#611620, Invitrogen/Life Technologies).

Validation	For western blot and immunofluorescence, all commercially available antibodies were used as validated by the manufacturer for their specific assay according to their data sheet. In addition, the staining were consistent with the predicted cellular localization of the protein. We have confirmed that the LC3 antibody does not give an LC3-II band in autophagy null cells.

Eukaryotic cell lines

Policy information about cell lines

Cell line source(s)	HeLa, SH-SY5Y, HEK-293T, Huh7, H4 cell lines and human adipose-derived Mesenchymal Stem Cells (MSC) (#PCS-500-011) were purchased from the American Type Culture Collection (ATCC). MCF10A cells were purchased from Horizon (#HD PAR-058).
Authentication	All cell lines were ordered from ATCC or Horizon with authentication using Short Tandem Repeat (STR) analysis.
Mycoplasma contamination	All the cell lines were regularly tested for mycoplasma contamination using EZ-PCR Mycoplasma Detection Kit (#20-700-20 from Biological Industries).
Commonly misidentified lines (See ICLAC register)	None.

Animals and other organisms

Policy information about studies involving animals; ARRIVE guidelines recommended for reporting animal research

Laboratory animals	(A) The autophagy reporter mRFP-GFP-LC3 mice<br>(B) Wild type C57Bl/6J mice<br><br>The autophagy reporter mRFP-GFP-LC3 reporter mouse line was housed in individually ventilated cages with free access to standard animal food chow and water, in a climate-controlled room with a 12 h light/dark cycle. This mouse line was generated in our lab as previously described. As in our earlier study, we used 6 to 7-weeks-old C57BL/6 male or female mice for food deprivation in vivo. The ratio of sexes of used mice was 1:1 for the 48 h fasting experiment. The number of the mice used for the experiments are indicated for each experiment in the figure legends (n=6). No inclusion or exclusion criteria were used. No significant differences of LC3-II levels between sexes were observed.
Wild animals	No wild animals were used in this study.
Field-collected samples	No Field-collected samples were used in this study.
Ethics oversight	All mouse procedures were performed in accordance with the UK Animals (Scientific Procedures) Act with appropriate Home Office Project and Personal animal licenses and with local Ethics Committee approval.

Note that full information on the approval of the study protocol must also be provided in the manuscript.

Flow Cytometry

Plots

Confirm that:

☒ The axis labels state the marker and fluorochrome used (e.g. CD4-FITC).

☒ The axis scales are clearly visible. Include numbers along axes only for bottom left plot of group (a 'group' is an analysis of identical markers).

☒ All plots are contour plots with outliers or pseudocolor plots.

☒ A numerical value for number of cells or percentage (with statistics) is provided.

Methodology

Sample preparation	Transfected HeLa cells were trypsinised and suspended in medium supplemented with DAPI
Instrument	Attune NxT Flow Cytometer (ThermoFisher Scientific)
Software	FlowJo v10 software
Cell population abundance	total cells: >80%, single cells: >95% , GFP+ cells: ~60% for GFP and ~30% for GFP-A53T, DAPI+;GFP+ cells: ~10-15%
Gating strategy	1st gate FSC-A/SSC-A -> 2nd gate FSC-A/FSC-H -> 3rd gate GFP+ cells -> 4th gate DAPI+;GFP+ cells. GFP+ and GFP

☒ Tick this box to confirm that a figure exemplifying the gating strategy is provided in the Supplementary Information.
